# Brasesquilignan A–E, Five New Furofurans Lignans from *Selaginella braunii* Baker

**DOI:** 10.3390/molecules27196349

**Published:** 2022-09-26

**Authors:** Fei Cheng, Jianping Wu, Yan Zhang, Yuyan Wang, Guihua Li, Hongliang Zeng, Xiaoai He, Guiming Deng, Jianbin Tan, Hongping Long, Puhua Zeng, Yiheng Liu, Gangzhi Zhu, Zuhui Chen, Kangping Xu

**Affiliations:** 1 Xiangya School of Pharmaceutical Sciences, Central South University, Changsha 410013, China; 2 Institute of Chinese MateriaMedica, Hunan Academy of Chinese Medicine, Changsha 410013, China; 3 Hunan QingYa Health Service Limited Company, Changsha 410083, China; 4 Haikou People’s Hospital and Central South University, Xiangya School of Medicine Affiliated Haikou Hospital, Haikou 570208, China; 5 The First Hospital of Hunan University of Chinese Medicine, Changsha 410007, China; 6 Hunan Key Laboratory of Diagnostic and Therapeutic Drug Research for Chronic Diseases, Central South University, Changsha 410013, China

**Keywords:** Selaginellaceae, *Selaginella braunii* Baker, furofurans lignans, anti-proliferativeactivity

## Abstract

Five new furofurans lignans, Brasesquilignan A–E (**1–5**), were isolated from the aqueous ethanol extract of *Selaginella braunii* Baker. Their structures were elucidated by extensive analysis of NMR and HRESIMS data. Their absolute configurations were determined by CD spectra, enzymatic hydrolysis, and GCMS analysis. Furthermore, all compounds were evaluated for anti-proliferative activities against various human cancer cellsin vitro. Compounds **2** and **3** exhibited weak inhibitorypotency against five human cancer cells.

## 1. Introduction

**Figure 1:**
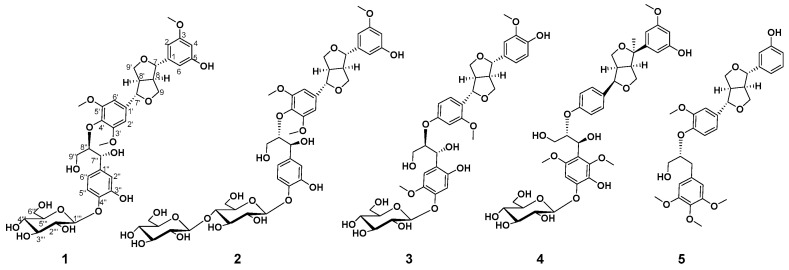
Structures of compounds **1**–**5**.

*Selaginella braunii* Baker is a perennial herb belonging to the genus *Selaginella* and mainly distributed south of the Yangtze River [1]. The whole plant is commonly used in traditional Chinese medicine forantiphlogistic, detoxicating, heat-clearing, and cough-relieving purposes. In previous phytochemical investigations of the genus *Selaginella*, lignans were a class of abundant chemical components, and diverse structural types of lignans have been isolated [2,3,4,5,6,7,8]. Lignans from *Selaginella* mainly consisted of sinapyl or piniol alcohol derivatives and mainly included neolignans, dibenzyltyrolactones, furofurans, norlignans, dibenzylbutanes, and oxyneolignans. Among them, the type of furofurans lignans was one of the most important. Modern pharmacological studies indicated its diverse bioactivities, such as antitumor [2], neuroprotective [3], and antioxidant [4] properties. As part of continuing research on the discovery of novel bioactive secondary metabolites from *Selaginella*, five new furofurans lignans, brasesquilignans A-E (**1**–**5**) (Figure 1), were obtained from the 75% EtOH extract of *S.braunii* Baker. Their structures, including absolute configuration, were elucidated by spectroscopic methods and enzymatic hydrolysis. Moreover, all compounds were evaluated for their anti-proliferative activities against various human cancer cells in vitro.

## 2. Results and Discussion

**Figure 2:**
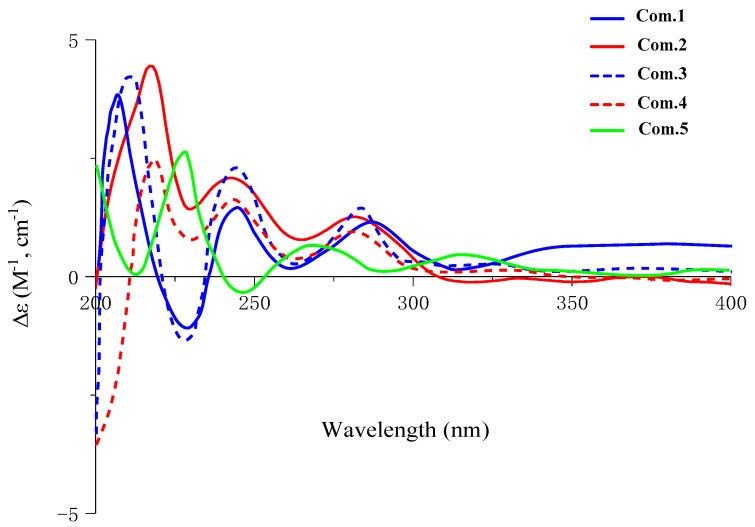
The CD spectra of compounds **1**–**5** (MeOH).

Compound **1** was obtained as white amorphous powder, and its molecular formula was confirmed asC_36_H_44_O_16_ by HRESIMS 755.2681 [M + Na]^+^ (calcd. for C_36_H_44_NaO_16_, 755.2527). The ^1^H NMR spectrum of **1** exhibited signals for eight aromatic protons, indicating the existence of one set of 1, 3, 5-trisubstituted benzene system(*δ*_H_ 7.03 (1H, brs, H-6), 6.87 (1H, brs, H-2), and 6.78 (1H, m, H-4)), one set of 1, 3, 4-trisubstituted aromatic proton signals (*δ*_H_ 6.97 (1H, d, *J* = 1.6 Hz, H-2″), 6.78 (1H, m, H-6″), and 6.73 (1H, m, H-5″)), and one set of the 1, 3, 4, 5-tetra-substituted benzene system (*δ*_H_ 6.89 (1H, d, *J* = 1.4 Hz, H-6′) and 6.75 (1H, m, H-2′)). Moreover, there was one anomeric proton signal at *δ*_H_ 4.25 (1H, d, *J* = 7.8 Hz, H-1‴), as well as three methoxyl proton signals at *δ*_H_ 3.79 (3H, brs, 3-OCH_3_), 3.76 (3H, brs, 3′-OCH_3_), and 3.76 (3H, brs, 5′-OCH_3_). The ^13^C NMR spectrum of **1** showed 30 carbon signals, of which *δ*_C_ 147.3, 119.2, 115.6, 111.0, 70.6, and 56.1 were overlapping signals. The ^1^H and ^13^C NMR (Table 1 and Table 2) indicated a furofuran lignan glycoside for **1**, which shared high similarity with those of *erythro*-syringylglycerol-*β*-*O*-4′-(+)-isoeucommin A 4‴-*O*-*β*-D-glucopyranoside [9], except for the different substitution of aryl groups with the C-4/C-5″replaced by hydrogen and C-5/C-3″ replaced by hydroxyl. The location of the substitution of aryl groups of **1** wasfurther determined by HMBC spectroscopic analysis (Figure 2).The small coupling constants of *J*_H-7, H-8_ (4.1Hz)/*J*_H-7′, H-8′_ (4.0 Hz) and the chemical shift differences of Δ*δ*_H–9_(0.4) and Δ*δ*_H–9′_(0.4) (Δ*δ*_H–9_ = *δ*_H–9a_–*δ*_H–9b_ andΔ*δ*_H–9′_ = *δ*_H–9′a_–*δ*_H–9′b_) showed that the relative configuration was *erythro* [10,11,12]. In addition, the coupling constant of *J*_H-7″, H-8″_, (7.4 Hz) confirmed the relative configuration as *threo* [13,14,15]. According to the CD spectrum of **1** (Figure 2), the positive Cotton effect at 285 nm and negative Cotton effect at 228 nm indicated that the absolute configuration was determined as 7*S*, 7′*S*, 8*R*, 8′*R*, 7″*R* and 8″*R* [16,17,18,19].The coupling constant of the anomeric proton (*δ*_H_4.89, d, *J* = 7.4, H-1‴) prompted the existence of *β*-configuration. The presence of D-glucose was confirmed by enzymatic hydrolysis and GC-MS analysis compared with authentic material. Thus, the structure of **1** was identified as (-) (7*S*, 7′*S*,7″*R*, 8*R*, 8′*R*, 8″*R*)-5, 3″-dihydroxy-3, 3′, 5′-trimethoxy-4″-*O*-*β*-D-glucopyranosyl-7, 9′: 7′, 9-diepoxy-4, 8″-oxy-8, 8′- sesquineolignan-7″, 9″-diol, named brasesquilignan **A** (Supplementary Materials).

**Figure 3:**
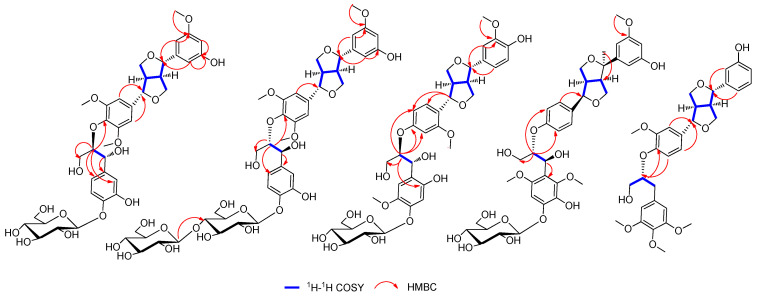
Key ^1^H-^1^H COSY and HMBC correlations of compounds **1**–**5**.

Compound **2** was obtained as a white amorphous powder, and its molecular formula was confirmed asC_42_H_54_O_21_, determined by HRESIMS 895.8730 [M + H]^+^ (calcd. for C_42_H_55_O_21_895.8730). Careful comparison of the NMR data of **2** (Table 1 and Table 2) with those of **1** revealed that the **2** was a glycoside of **1** located at C-4‴, which was confirmed by HMBC (Figure 3) correlations between anomeric proton H-1″″(*δ*_H_4.25, d, *J* = 7.7)and C-4‴ (*δ*_C_ 70.4). The two sugars were confirmed as *β*-D-configuration by the anomeric protons coupling constant (*δ*_H_4.24, d, *J* = 7.7, H-1‴and *δ*_H_4.25, d, *J* = 7.7, H-1″″), further enzymatic hydrolysis, and GC-MS analysis. The similar coupling constants (*J*_H-7, H-8_, *J*_H-7′, H-8′_, and *J*_H-7″, H-8″_)of **2** showed the relative configuration was consistent with **1.** However, the CD spectrum of **2** (Figure 2) showed a positive Cotton effect at 228 nm and 285nm, illustrating that the absolute configuration of **2** was 7*S*, 7′*S*,7″*S*, 8*R*, 8′*R*, 8″*S*. Therefore, the structure of **2** was identified as (+) (7*S*, 7′*S*,7″*S*, 8*R*, 8′*R*, 8″*S*)-5, 3″-dihydroxy-3, 3′, 5′-trimethoxy-4″-*O-β*-D-glucopyranosyl-(1→4)-*O-β*-D-glucopyranosyl-7, 9′: 7′, 9- diepoxy-4, 8″-oxy-8, 8′-sesquineolignan-7″, 9″-diol, named brasesquilignan **B**.

Compound **3** was obtained as a white amorphous powder, and its molecular formula was confirmed asC_36_H_44_O_16_by HRESIMS 755.2473 [M + Na]^+^(calcd. for C_36_H_44_NaO_16_, 755.2527). The ^1^H NMR and ^13^C NMR data (Table 1 and Table 2) of **3** were quite similar to those of **1,** except for the different substitution of aryl groups. The ^1^H NMR of **3** indicated the existence of a set of 1, 3, 4-trisubstituted aromatic proton signals, *δ*_H_ 6.88 (1H, d, *J* = 1.6 Hz, H-2), 6.75 (1H, d, *J* = 1.6 Hz, H-6), and 6.72 (1H, d, *J* = 8.1 Hz, H-5); a set of 1, 2, 4-trisubstituted aromatic proton signals, *δ*_H_ 7.06 (1H, d, *J* = 8.5 Hz, H-6′), 6.97 (1H, d, *J* = 1.9 Hz, H-3′), and 6.85 (1H, dd, *J* = 8.6, 1.8 Hz, H-5′); and a set of 1, 3, 4, 6-tetra-substituted aromatic proton signals, *δ*_H_ 6.87 (1H, brs, H-5″) and 6.86 (1H, brs, H-2″). The location of the functional groups and NMR data assignments of **3** were determined by HMBC and HSQC spectroscopic analysis (Figure 2). The sugar of **3** was confirmed as *β*-D-configuration by the anomeric proton coupling constant (*δ*_H_4.89, d, *J* = 7.4, H-1‴), enzymatic hydrolysis, and GC-MS analysis. Comparing coupling constants (*J*_H-7, H-8_, *J*_H-7′, H-8′_, and *J*_H-7″, H-8″_) and CD spectra of **3** with **1** (Figure 2), the absolute configuration of **3** was determined as7*S*, 7′*S*,7″*R*, 8*R*, 8′*R*, 8″*R*. Therefore, the structure of **3** was identified as (-) (7*S*, 7′*S*, 7″*R*, 8*R*, 8′*R*, 8″*R*)-4, 2″- dihydroxy-3, 2′, 5″-trimethoxy-4″-*O*-*β*-D-glucopyranosyl-7, 9′: 7′, 9-diepoxy-4, 8″-oxy-8, 8′- sesquineolignan-7″, 9″-diol, named brasesquilignan **C**.

Compound **4** was obtained as a white amorphous powder, which had a molecular formula of C_37_H_46_O_14_ based on a protonated molecular ion peak at *m*/*z* 769.2638 [M + Na]^+^ (calcd. for C_37_H_46_NaO_14_ 769.2684) in the HRESIMS data. The ^1^H NMR and ^13^C NMR data (Table 1 and Table 2) suggested that **4** and **1** are the same type of compound. However, the ^1^H NMR of compound **4** showed eight aromatic proton signals, including a set of 1, 4-disubstituted aromatic proton signals, *δ*_H_ 6.97 (1H, d, *J* = 1.5 Hz, H-5′), 6.82 (2H, brs, H-2′), 6.78 (1H, dd, *J* = 8.0, 1.6 Hz, H-3′), and 6.70 (3H, m, H-6′); a set of 1, 3, 5-trisubstituted aromatic proton signals, *δ*_H_ 6.82 (2H, brs, H-6) and 6.70 (3H, m, H-2/4); and a 1, 2, 3, 4, 6-penta-substituted aromatic proton signal, *δ*_H_ 6.74 (1H, brs, H-5″). In addition, compound **4** had one more methyl proton signal at *δ*_H_ 1.07 (3H, brs, 7-CH_3_). In the ^13^C NMR spectrum of **4**, the carbon signals *δ*_C_ 67.5, 52.9, 79.6,and 22.0 were significantly different from **1**, and *δ*_C_ 79.6 is a quaternary carbon, suggesting that there is a methyl on the furan ring of **4**. Further, the correlation between *δ*_H_6.82 (2H, brs) and *δ*_H_6.78 (1H, dd, *J* =1.6, 8.0 Hz) in the ^1^H-^1^H COSY spectrum and the HMBC (Figure 3) correlations from H-2′ (*δ*_H_6.82, 1H, brs) to C-7′ (*δ*_C_ 82.3), H-3′ (*δ*_H_6.78, 1H, dd, *J* = 1.6, 8.0 Hz) to C-8″ (*δ*_C_ 87.3),and 7-CH_3_ (*δ*_H_ 1.07, 3H,) to C-8 (*δ*_C_ 67.5) indicated that the methyl was located at C-7 (Figure 3). The HMBC (Figure 3) correlations between H-1‴(*δ*_H_4.23, 1H, d, *J* = 7.8 Hz) and C-4″ (*δ*_C_132.4) indicated that the glycosyl was located at C-4″. The coupling constant of the anomeric protons (*J* = 7.8 Hz) prompted the existence of *β*-configuration. Moreover, the presence of D-glucose was confirmed by enzymatic hydrolysis and GC-MS analysiscompared with standard material. The coupling constants (*J*_H-7′, H-8′_ = 6.4 Hz and *J*_H-7″, H-8″_= 7.2 Hz) showed that the relative configurations were both *threo*. The CD spectrum of **4** (Figure 2) showed a positive Cotton effect at both 285nm and 240nm, illustrating that the absolute configuration was 7*S*, 7′*S*,7″*S*, 8*R*, 8′*R*, 8″*S*. Therefore, the structure of **4** was identified as (-) (7*S*, 7′*S*,7″*S*, 8*R*, 8′*R*, 8″*S*)-5, 3″-dihydroxy-3, 2″, 6″-trimethoxy-4″-*O*-*β*-D-glucopyranosyl-7, 9′: 7′, 9-diepoxy-7-methyl-4, 8″-oxy-8, 8′-sesquineolignan-7″, 9″-diol, named brasesquilignan **D**.

Compound **5** was obtained as a white amorphous powder. Its molecular formula was determined to be C_31_H_36_O_9_by HRESIMS at *m/z* 553.2416 [M + H]^+^ (calcd. for C_31_H_37_O_9_ 553.2438). Compound **5** had a similar ^1^H and ^13^C spectrum (Table 1 and Table 2) to compound **1**, except that there were no sugar-related signals and different substituent positions. The ^1^H NMR spectrum showed nine aromatic protons, including a group of 1, 3-disubstituted aromatic proton signals, *δ*_H_ 6.89 (1H, d, *J* = 1.4 Hz, H-2), 6.76 (1H, d, *J* = 1.6 Hz, H-6), 6.74 (1H, d, *J* = 1.6 Hz, H-4), and 6.59 (1H, m, H-5); one set of 1, 3, 4-trisubstituted aromatic proton signals, *δ*_H_ 6.65 (1H, d, *J* = 8.0 Hz, H-5′) and 6.60 (2H, brs, H-2′ / 6′); and a set of 1, 3, 4, 5-tetrasubstituted aromatic proton signals, *δ*_H_ 6.73 (1H, brs, H -2″) and 6.71 (1H, brs, H-6″). The ^13^C NMR spectrum showed18 aromatic carbon signals, *δ*_C_ 104.1~153.6; 4 methoxy carbon signals, *δ*_C_ 56.5 (5″-OCH_3_), 56.4 (4″-OCH_3_), 56.1 (3″-OCH_3_), and 56.0 (3′-OCH_3_); and 9 aliphatic carbon signals. In the HMBC spectrum, the correlation between H-5′ (*δ*_H_ 6.65, 1H, d, *J* = 8.0 Hz,) with C-8″ (*δ*_C_ 84.0) revealed that the connection of the two fragments is C-4′-*O*-C-8″. The location of the other functional groups and NMR data assignments of **5** were determined by HMBC and HSQC spectroscopic analysis (Figure 3). The relative configuration of **5** was determined by the chemical shift differences ofthe two pairs of diastereotopic methylene protons of H-9 and H-9′. The approximately equal values of Δ*δ*_H–9_(0.4) and Δ*δ*_H–9′_(0.4) suggested thatH-7/H-8 and H-7′/H-8′ were*Trans* [10,12]. The CD spectrum of **5** (Figure 2) showed the positive Cotton effect at both 280 nm and 230 nm, illustrating that the absolute configuration was 7*S*, 7′*S*,7″*S*, 8*R*, 8′*R*, 8″*S.* Therefore, the structure of **5** was identified as (+)(7*S*, 7′*S*,7″*S*, 8*R*, 8′*R*, 8″*S*) 5, 3″-dihydroxy -3, 3′, 5′-trimethoxy-7, 9′: 7′, 9-diepoxy-4, 8″-oxy-8, 8′-sesquineolignan-9″-alcohol, named brasesquilignan **E**.

All compounds were examined for their anti-proliferative activity on A375, A549, MCF-7, MDA-MB-231,and SK-MEL-28 cells by the MTT assay using standard staurosporine (STS) as a positive control. All compounds exhibited weak inhibitory potency againstA549 and MCF-7cells (Table 3).

## 3. Materials and Methods

### 3.1. General Experimental Procedures

HRESIMS data were measured on an Agilent Technologies liquid chromatograph connected to Q-TOF mass spectra (Thermo Fisher, Massachusetts, MA, USA). NMR spectra were recorded on a Bruker AV-500 MHz spectrometer (Bruker, Karlsruhe, Germany) using DMSO-*d*_6_ as solvent and tetramethylsilane (TMS).GCMS was measured on GCMS-QP2010 Ultra (Shimadzu Corporation, Kyoto, Japan). Column chromatography (CC) was performed on HW-40C (TOYOPEARL TOSOH, Tokyo, Japan). Optical rotations were measured on an INESA SGW-3 polarimeter. Analytical and Semi-preparative HPLC was performed on an Agilent 1200 equipped with a DAD detector and a siligreen C18 column (5/10 μm, 250 × 10 mm, siligreen, Beijing, China). All solvents were of analytical grade.

### 3.2. Plant Material

The whole herbs of *S.braunii* Baker were collected from Hunan province in People’s Republic of China, in August 2015, and identified by Prof. Kangping Xu (Xiangya School of Pharmaceutical Sciences, Central South University). A specimen (no. 20150816) was deposited at the Xiangya School of Pharmaceutical Sciences, Central South University.

### 3.3. Extraction and Isolation

Whole herbs of *S.braunii* Baker (13.0 kg) were exhaustively extracted with 75% aqueous ethanol under reflux (2 times, 104 L × 2 h).After vacuum concentration, the extract was suspended in H_2_O and partitioned with petroleum ether, ethyl acetate. The water fraction (200 g) was fractionated by Macroporous resin HPD-100column chromatography, successively eluting with H_2_O, 30, 70, and 95% EtOH-H_2_O to obtain four fractions (FrA-D). FrC was performed on HW-40C (MeOH/H_2_O in gradient) to obtain seven fractions (FrC_1_-C_7_). FrC_3_ was subjected to gel column chromatography and semi-preparative liquid chromatography (3.0 mL/min, 280 nm, ACN-H_2_O, 3.0:7.0, *V*/*V*) repeatedly to obtain compounds1 (2.0 mg), 3 (2.6 mg), and 4 (1.0 mg). FrC_2_ was subjected to gel column chromatography and semi-preparative HPLC (3.0 mL/min, 280 nm, ACN-H_2_O, 3.0:7.0, V/V) repeatedly to obtain compound 2 (1.5 mg). FrC_4_was further purified by repeated chromatography (gel column and semi-preparative RP-HPLC) to yield compound 5 (2.9 mg).

Brasesquilignan A (**1**): white amorphous powder, ${\left[\alpha \right]}_{D}^{25}$-30.2 (*c* 0.06, MeOH), HPLC-UV (ACN-H_2_O) *λ*_max_nm: 205, 225, 280, HRESIMS, *m*/*z* 755.2681 [M + Na]^+^ (calcd. for C_36_H_44_NaO_16_755.2527),^1^H NMR (500 MHz in DMSO-*d*_6_) and ^13^C NMR (125 MHz in DMSO-*d*_6_). For data, see Table 1 and Table 2.

Brasesquilignan B (**2**): white amorphous powder, ${\left[\alpha \right]}_{D}^{25}$-2.3 (*c* 0.05, MeOH), HPLC-UV (ACN-H_2_O) *λ*_max_ nm: 205, 225, 280, HRESIMS, *m*/*z*: 895.8730 [M+H]^+^ (calcd. for C_42_H_55_O_21_895.8730),^1^H NMR (500 MHz in DMSO-*d*_6_) and ^13^C NMR (125 MHz in DMSO-*d*_6_).For data, see Table 1 and Table 2.

Brasesquilignan C (**3**): white amorphous powder, ${\left[\alpha \right]}_{D\;}^{25}$-10.4 (*c* 0.2, MeOH), HPLC-UV (ACN-H_2_O) *λ*_max_ nm: 205, 225, 280, HRESIMS, *m*/*z*: 755.2473 [M+Na]^+^(calcd. for C_36_H_44_NaO_16_755.2527),^1^H NMR (500 MHz in DMSO-*d*_6_) and ^13^C NMR (125 MHz in DMSO-*d*_6_).For data, see Table 1 and Table 2.

Brasesquilignan D (**4**): white amorphous powder, ${\left[\alpha \right]}_{D\;}^{25}$-15.4 (c 0.05, MeOH), HPLC-UV (ACN-H_2_O) λ_max_ nm: 205, 225, 280, HRESIMS, *m*/*z*: 769.2638 [M+Na]^+^ (calcd. for C_37_H_46_NaO_14_, 769.2684),^1^H NMR (500 MHz in DMSO-d_6_) and ^13^C NMR (125 MHz in DMSO-d_6_). For data, see Table 1 and Table 2.

Brasesquilignan E (**5**): white amorphous powder, ${\left[\alpha \right]}_{D\;}^{25}$+28.2 (*c* 0.05, MeOH), HPLC-UV (ACN-H_2_O) *λ*_max_ nm: 205, 225, 280, HRESIMS, *m*/*z*: 553.2416 [M+H]^+^ (calcd. for C_31_H_37_O_9_, 553.2438), ^1^H NMR (500 MHz in DMSO-*d*_6_) and ^13^C NMR (125 MHz in DMSO-*d*_6_). For data, see Table 1 and Table 2.

### 3.4. Enzymatic Hydrolysis

Compounds **1**–**5** (each 0.5 mg), cellulase (400 u/mg), and buffered saline solution (acetic acid/sodium acetate, PH = 6, 1 mL) were added in centrifuge tube (5 mL, sample: cellulose= 1:30), and incubated for 96 h in 37 °C. After extraction with CHCl_3_, the aqueous layer of reaction mixture was concentrated and dried to obtain the monosaccharide fraction. The residue was dissolved in pyridine (0.6 mL) with 1.0 mg of L-cysteine methyl ester hydrochloride and heated (60 °C, 1 h). Then, trimethylsilylimidazole (0.6 mL) was added and heated (60 °C, 1 h). The reaction mixture was analyzed by GC-MS under the following conditions: Column, Rtx-5MS (0.5 μm × 30.0 mm, 0.32 mm); front inlet 300 °C, column 150–300 °C at 15 °C/min. The monosaccharides of compounds were confirmed by comparing the retention times with those of standard sugar (subjected to the same method).

### 3.5. Anti-Proliferative Evaluation

All compounds were tested with positive control (staurosporine, STS). MDA-MB-231, SK-MEL-28, and A375 (Shanghai Institutes for Biological Sciences, Chinese Academy of Sciences) were cultured in DMEM/F12 (1:1) medium (Hyclone) supplemented with 10% FBS and 100 U/mL penicillin/streptomycin at 37 °C and 5% CO_2_. MCF-7 and A549 (Shanghai Institutes for Biological Sciences, Chinese Academy of Sciences) were cultured by DMEM/HIGH GLUCOSE medium (Hyclone) supplemented with 10% FBS and 100 U/mL penicillin/streptomycin at 37 °C and 5% CO_2_. All human cancer cells were plated in 96-well plates (25,000 cells/mL). Then, each well was supplemented at various concentrations of test compounds in triplicate for 24 h, and MTT (20 μL 5 mg/mL) was added. After incubation for 4 h, the medium was removed, and DMSO (150 μL) was added before further incubation (30 min, 37 °C). The OD (570 nm) value of each well was measuredwitha microplate reader MD5 (Molecular devices, San Jose, CA, USA). The half-inhibitory concentration (IC_50_) values were calculated by SPSS 25. software.

## Figures and Tables

**Table 1:** ^1^H NMR (500 MHz) data of **1**–**5** in DMSO-*d*_6_.

Position	1	2	3	4	5
1					
2	6.87, brs	6.86, brs	6.88, d (1.6)	6.70, m	6.89, d (1.4)
3					
4	6.78, m	6.78, m		6.70, m	6.74, d (1.6)
5			6.72, d (8.1)		6.59, m
6	7.03, brs	7.03, brs	6.75, d (1.6)	6.82, brs	6.76, d (1.6)
7	4.63, d (4.1)	4.62, d (4.0)	4.63, t (4.2)		4.60, m
8	3.06, m	3.05, m	3.06, m	3.47 overlapped	3.03, m
9a	4.13, m	4.15, m	4.13, m	3.88, m	4.13, m
9b	3.73, m	3.72, m	3.73, m	3.57, m	3.73, m
1′					
2′	6.75, m	6.73, m		6.82, brs	6.60, brs
3′			6.97, d (1.9)	6.78, dd (1.6, 8.0)	
4′					
5′			6.85, dd (8.6, 1.8)	6.97, d (1.5)	6.65, d (8.0)
6′	6.89, d (1.4)	6.88, brs	7.06, d (8.5)	6.70, m	6.60, brs
7′	4.61, d (4.1)	4.64, d (4.0)	4.63, t (4.2)	4.63, d (6.4)	4.60, m
8′	3.06, m	3.05, m	3.06, m	2.19, m	3.03, m
9′a	4.13, m	4.15, m	4.13, m	3.68, m	4.13, m
9′b	3.73, m	3.72, m	3.73, m	3.46 overlapped	3.73, m
1″					
2″	6.97, d (1.6)	6.96, brs			6.73, brs
3″			6.87, brs		
4″					
5″	6.73, m	6.75, m		6.74, brs	
6″	6.78, m	6.78, m	6.86, brs		6.71, brs
7″	3.61, m	3.58, m	3.43, m	3.59, m	4.11, m3.40 overlapped
8″	5.47, d (7.4)	5.45, d (7.4)	5.51, d (6.5)	5.46, d (7.2)	4.12, m
9″a	3.97, m	3.72, m	3.72, m	3.95, m	4.11, m
9″b	3.72, m	3.04, m	3.63, m	3.06, m	3.40 overlapped
1‴	4.25, d (7.8)	4.24, d (7.7)	4.89, d (7.4)	4.23, d (7.8)	
2‴	3.00, m	2.97, m	3.25, m	3.00, m	
3‴	3.08, m	3.05, m	3.25, m	3.11, m	
4‴	3.06, m	3.06, m	3.14, m	3.06, m	
5‴	3.18, m	3.16, m	3.26, m	3.10, m	
6‴	3.65, m 3.42, overlapped	3.65, m 3.42, overlapped	3.64, m 3.43, m	3.66, m 3.43 overlapped	
–OCH_3_	(3-) 3.79, brs	(3-) 3.78, brs	(4-) 3.77, brs	(3-) 3.76, brs	(3″-) 3.71, brs
	(3′-) 3.76, brs	(3′-) 3.76, brs	(2′-) 3.75, brs	(2″-) 3.74, brs	(3′-) 3.74, brs
	(5′-) 3.76, brs	(5′-) 3.75, brs	(3″-) 3.80, brs	(6″-) 3.74, brs	(4″-) 3.75, brs
					(5″-) 3.76, brs
7-CH_3_				1.07, d (6.7)	
1″″		4.25, d (7.7)			
2″″		2.98, m			
3″″		3.05, m			
4″″		3.05, m			
5″″		3.33, m			
6″″		3.50 overlapped			

**Table 2:** ^13^C NMR (125 MHz) data of **1**–**5** in DMSO-*d*_6_.

Position	1	2	3	4	5
1	135.2	135.2	132.6	134.5	145.1
2	111.0	110.9	110.9	113.2	110.9
3	144.0	144.0	148.0	143.9	146.4
4	119.2	119.2	146.4	115.5	119.1
5	147.3	147.3	115.6	146.0	122.1
6	115.6	115.6	119.0	117.6	114.1
7	85.9	85.9	85.5	79.6	85.6
8	54.3	54.2	54.0	67.5	54.0
9	71.5	71.4	71.5	72.3	71.4
1′	146.6	146.4	146.6	135.1	135.3
2′	115.6	115.7	149.5	110.4	104.1
3′	148.0	148.0	110.9	119.1	148.0
4′	132.0	132.1	135.7	146.1	129.8
5′	148.1	148.1	118.3	110.9	115.6
6′	111.0	110.9	115.6	118.7	104.1
7′	85.6	85.6	85.9	82.3	85.8
8′	54.0	54.0	54.3	52.9	54.1
9′	71.3	71.4	71.5	59.0	71.5
1″	129.5	129.5	129.4	129.2	129.1
2″	111.1	110.9	147.3	147.8	115.6
3″	147.3	147.3	115.6	146.8	148.4
4″	132.5	132.6	135.0	132.4	153.6
5″	115.7	115.6	144.0	115.6	148.4
6″	119.2	119.1	110.9	148.0	104.1
7″	51.0	51.0	53.9	50.9	61.9
8″	87.4	87.2	87.2	87.3	84.0
9″	70.6	70.6	63.4	70.5	62.2
1‴	103.3	103.2	100.5	103.2	
2‴	73.9	73.9	73.6	73.9	
3‴	77.3	77.2	77.3	77.2	
4‴	70.6	70.4	70.1	70.5	
5‴	77.4	77.3	77.5	77.4	
6‴	61.5	61.5	61.0	61.5	
-OCH_3_	(3-) 56.2	(3-) 56.2	(4-) 56.2	(3-) 56.1	(3″-) 56.1
	(3′-) 56.1	(3′-) 56.1	(2′-) 56.2	(2″-) 56.0	(3′-) 56.0
	(5′-) 56.1	(5′-) 56.1	(3″-) 56.1	(6″-) 56.0	(4″-) 56.4
					(5″-) 56.5
7-CH_3_				22.0	
1″″		103.9			
2″″		73.8			
3″″		77.2			
4″″		68.9			
5″″		76.3			
6″″		67.5			

**Table 3:** Anti-proliferative activity ofall compoundsagainst five human cancer cell linesin vitro.

Compound	IC_50_ (μM)				
	SK-MEL-28	A375	A549	MCF-7	MDA-MB-231
**1**	N/A	N/A	N/A	93.69 ± 5.54	N/A
**2**	48.30 ± 5.29	35.12 ± 2.54	27.82 ± 2.38	22.09 ± 2.39	44.02 ± 2.32
**3**	56.82 ± 4.83	63.57 ± 1.49	38.88 ± 2.85	31.26 ± 1.14	53.56 ± 1.44
**4**	N/A	N/A	N/A	N/A	N/A
**5**	N/A	N/A	N/A	N/A	N/A
STS	0.04 ± 0.008	0.06 ± 0.006	0.4 ± 0.11	0.2 ± 0.04	0.03 ± 0.005

N/A: Not active; STS: Staurosporine used as a positive control.

## Data Availability

The data presented in this study are available in the Supplementary Materials.

## Supplementary Materials

The following supporting information can be downloaded at: https://www.mdpi.com/article/10.3390/molecules27196349/s1.

## Sample Availability

Samples of the compounds are available from the authors.
