# Supplementary material for: Brasesquilignan A–E, Five New Furofurans Lignans from Selaginella braunii Baker
Source: Molecules. 2022 Sep 26;27(19):6349. doi: 10.3390/molecules27196349 (PMC9572557; doi:10.3390/molecules27196349)
Supplement: Supplementary file 1 [file molecules-27-06349-s001.zip › molecules-1898881-supplementary.pdf]

rasesquilignan A-E, five new furofurans lignans from *Selaginella braunii* Baker

Fei Cheng [1,2], Jianping Wu [1,5], Yan Zhang [1], Yuyan Wang [1,5], Guihua Li [1,5], Hongliang Zeng [2], Xiaoai He [3], Guiming Deng [4], Jianbin Tan [1,6], Hongping Long [4], Puhua Zeng [2], Yiheng Liu [3], Gangzhi Zhu [3], Zuhui Chen [5], Kangping Xu [1,4,5,6*]

[1]*Xiangya School of Pharmaceutical Sciences, Central South University, Changsha, 410013, China*

[2]*Institute of Chinese Materia Medica, Hunan Academy of Chinese Medicine, Changsha, 410013, China*

[3]*Haikou People's Hospital and Central South University, Xiangya School of Medicine Affiliated Haikou Hospital, Haikou, 570208, China*

[4]*The First Hospital of Hunan University of Chinese Medicine, Changsha, 410007, China*

[5]*Hunan QingYa Health Service Limited Company, Changsha, 410000, China*

[6]*Hunan Key laboratory of Diagnostic and Therapeutic Drug Research for Chronic Diseases, Central South University, Changsha, 410013, Changsha, China*

[*]Corresponding authors: Kangping Xu (K.P. X)

E-mail addresses: xukp395@csu.edu.cn

Contents

Figure S1. HRESIMS spectrum of compound **1** 4

Figure S2. ^{1}H NMR spectrum of compound **1** (DMSO-d_6, 500MHz) 4

Figure S3. ^{13}C NMR spectrum of compound **1** (DMSO-d_6, 125MHz) 5

Figure S4. DEPT135 spectrum of compound **1** (DMSO-d_6, 125MHz)........ 5

Figure S5. ^{1}H-^{1}H COSY spectrum of compound **1** (DMSO-d_6, 500MHz) 6

Figure S6. HSQC spectrum of compound **1** (DMSO-d_6, 500MHz)........ 6

Figure S7. HMBC spectrum of compound **1** (DMSO-d_6, 500MHz)........ 7

Figure S8. NOESY spectrum of compound **1** (DMSO-d_6, 500MHz) 7

Figure S9. CD spectrum of compound **1** (in MeOH) 8

Figure S10. GC-MS spectrum of compound **1**........ 8

Figure S11. HRESIMS spectrum of compound **2** 9

Figure S12. ^{1}H NMR spectrum of compound **2** (DMSO-d_6, 500MHz) 9

Figure S13. ^{13}C NMR spectrum of compound **2** (DMSO-d_6, 125MHz) 10

Figure S14. DEPT135 spectrum of compound **2** (DMSO-d_6, 125MHz)........ 10

Figure S15. ^{1}H-^{1}H COSY spectrum of compound **2** (DMSO-d_6, 500MHz) 11

Figure S16. HSQC spectrum of compound **2** (DMSO-d_6, 500MHz)........ 11

Figure S17. HMBC spectrum of compound **2** (DMSO-d_6, 500MHz)........ 12

Figure S18. NOESY spectrum of compound **2** (DMSO-d_6, 500MHz) 12

Figure S19. CD spectrum of compound **2** (in MeOH) 13

Figure S20. GC-MS spectrum of compound **2**........ 13

Figure S21. HRESIMS spectrum of compound **3** 14

Figure S22. ^{1}H NMR spectrum of compound **3** (DMSO-d_6, 500MHz) 14

Figure S23. ^{13}C NMR spectrum of compound **3** (DMSO-d_6, 125MHz) 15

Figure S24. DEPT135 spectrum of compound **3** (DMSO-d_6, 125MHz)........ 15

Figure S25. ^{1}H-^{1}H COSY spectrum of compound **3** (DMSO-d_6, 500MHz) 16

Figure S26. HSQC spectrum of compound **3** (DMSO-d_6, 500MHz)........ 16

Figure S27. HMBC spectrum of compound **3** (DMSO-d_6, 500MHz)........ 17

Figure S28. NOESY spectrum of compound **3** (DMSO-d_6, 500MHz) 17

Figure S29. CD spectrum of compound **3** (in MeOH) 18

Figure S30. GC-MS spectrum of compound **3** 18

Figure S31. HRESIMS spectrum of compound **4** 19

Figure S32. CD spectrum of compound **4** (in MeOH) 19

Figure S33. ^{1}H NMR spectrum of compound **4** (DMSO- d_6, 500MHz) 20

Figure S34. ^{13}C NMR spectrum of compound **4** (DMSO-d_6, 125MHz) 20

Figure S35. ^{1}H-^{1}H COSY spectrum of compound **4** (DMSO-d_6, 500MHz) 21

Figure S36. HSQC spectrum of compound **4** (DMSO-d_6, 500MHz) 21

Figure S37. HMBC spectrum of compound **4** (DMSO-d_6, 500MHz) 22

Figure S38. NOESY spectrum of compound **4** (DMSO-d_6, 500MHz) 22

Figure S39. GC-MS spectrum of compound **4** 23

Figure S40. HRESIMS spectrum of compound **5** 23

Figure S41. CD spectrum of compound **5** (in MeOH) 24

Figure S42. ^{1}H NMR spectrum of compound **5** (DMSO-d_6, 500MHz) 24

Figure S43. ^{13}C NMR spectrum of compound **5** (DMSO-d_6, 125MHz) 25

Figure S44. DEPT135 spectrum of compound **5** (DMSO-d_6, 125MHz) 25

Figure S45. ^{1}H-^{1}H COSY spectrum of compound **5** (DMSO-d_6, 500MHz) 26

Figure S46. HSQC spectrum of compound **5** (DMSO-d_6, 500MHz) 26

Figure S47. HMBC spectrum of compound **5** (DMSO-d_6, 500MHz) 27

Figure S48. NOESY spectrum of compound **5** (DMSO-d_6, 500MHz) 27

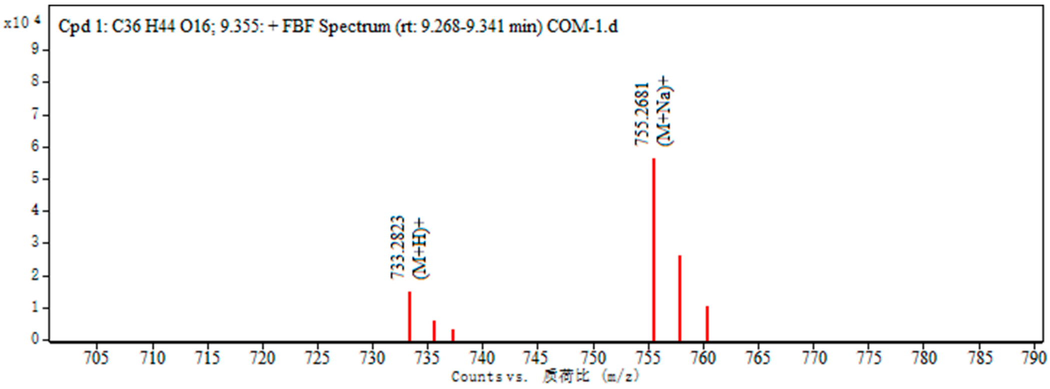

Figure S1. HRESIMS spectrum of compound **1**

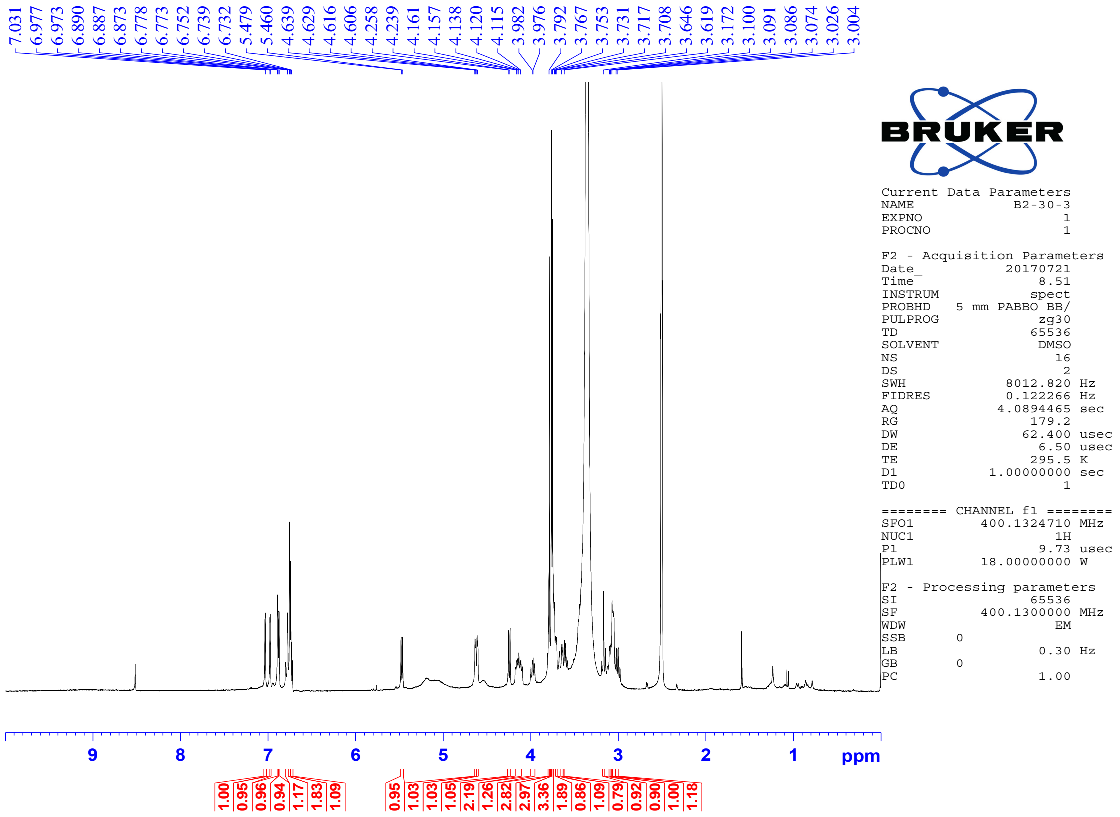

Figure S2. ^{1}H NMR spectrum of compound **1** (DMSO-d_6, 500MHz)

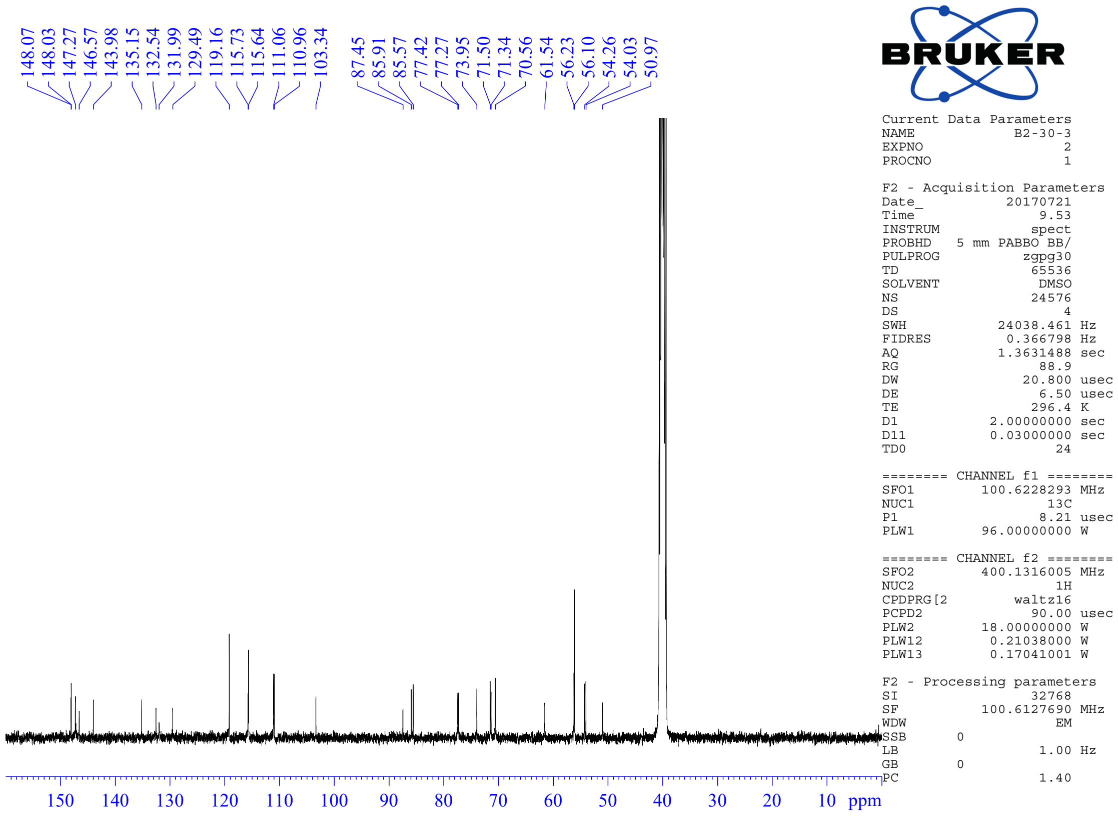

Figure S3. ^{13}C NMR spectrum of compound **1** (DMSO-d_6, 125MHz)

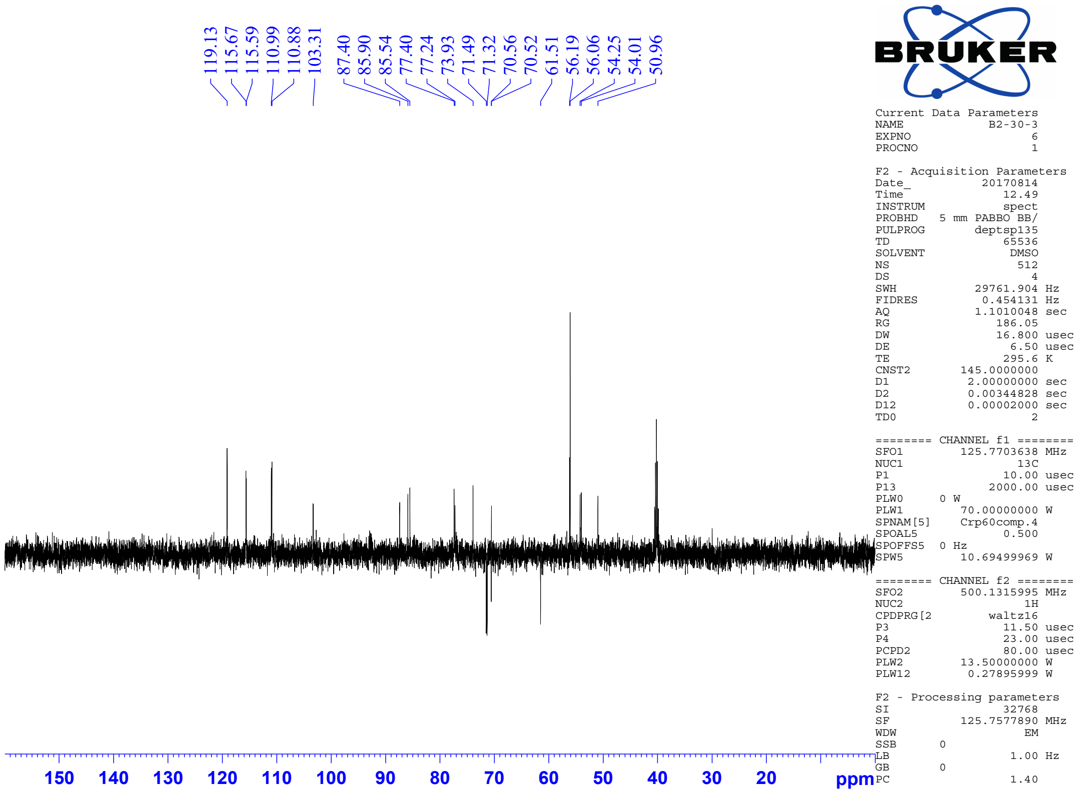

Figure S4. DEPT135 spectrum of compound **1** (DMSO-d_6, 125MHz)

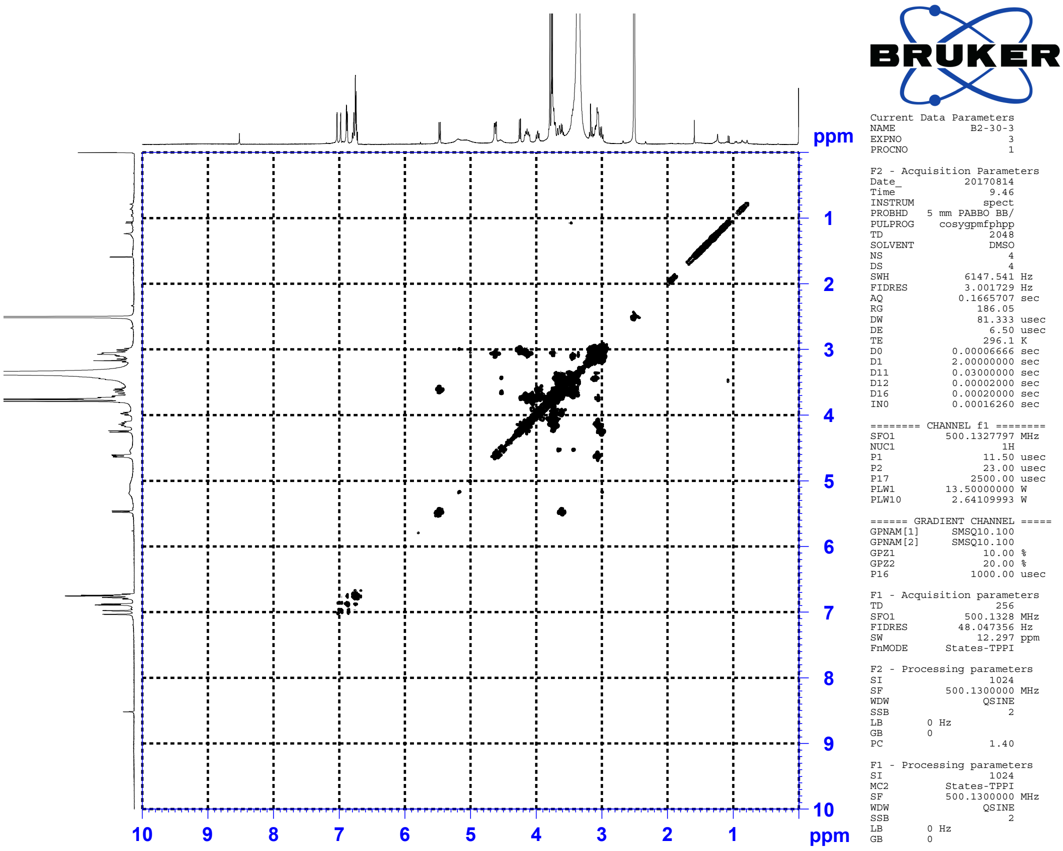

Figure S5. ^{1}H-^{1}H COSY spectrum of compound **1** (DMSO-d_6, 500MHz)

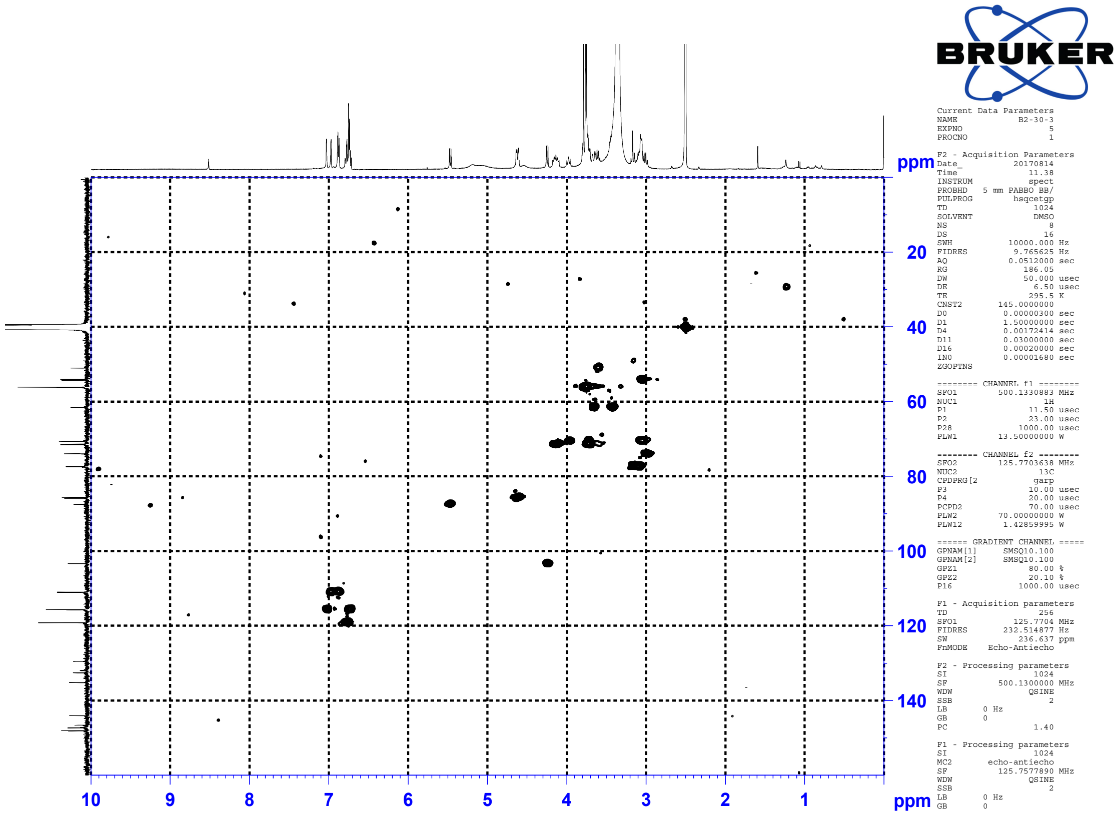

Figure S6. HSQC spectrum of compound **1** (DMSO-d_6, 500MHz)

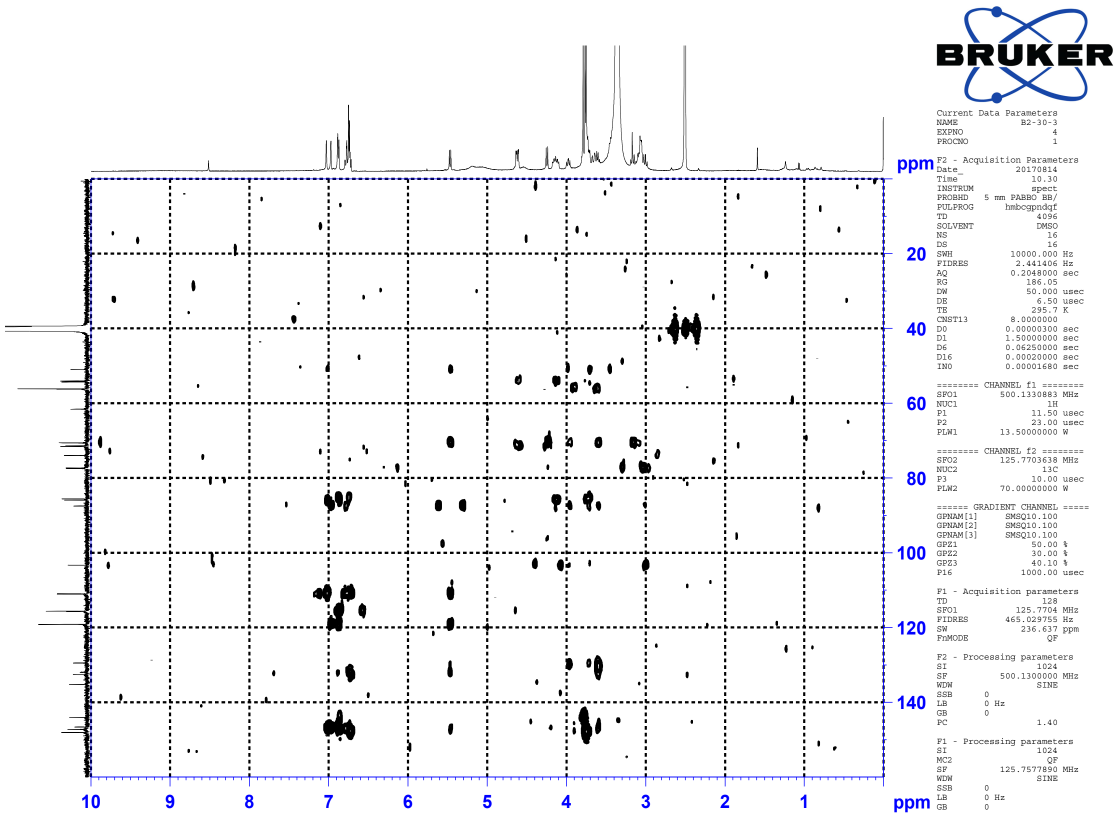

Figure S7. HMBC spectrum of compound **1** (DMSO-d_6, 500MHz)

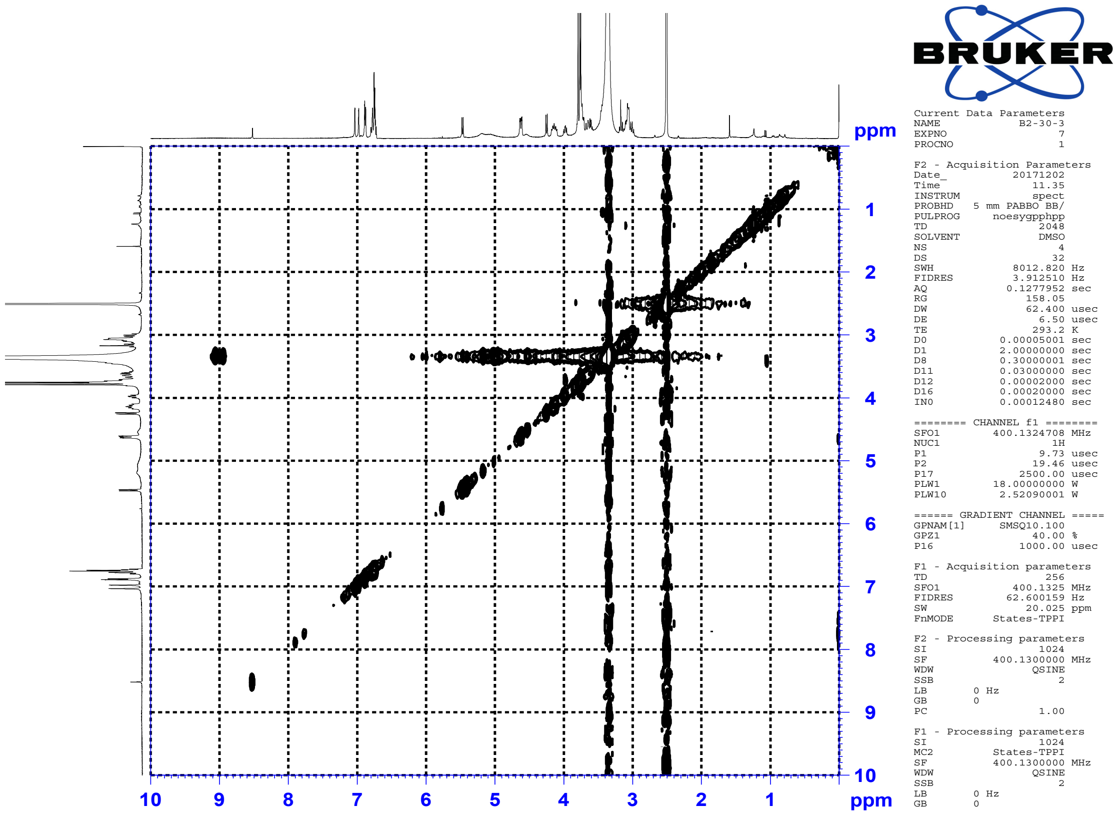

Figure S8. NOESY spectrum of compound **1** (DMSO-d_6, 500MHz)

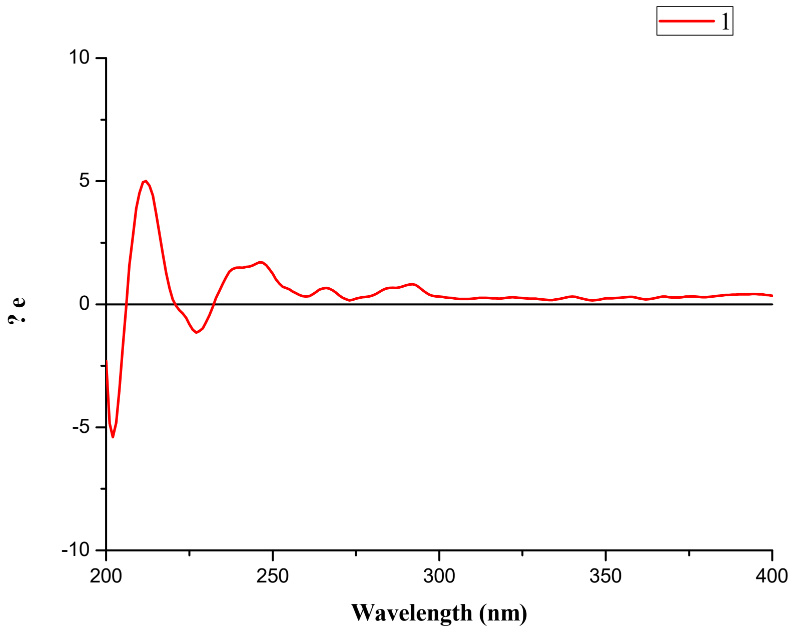

Figure S9. CD spectrum of compound **1** (in MeOH)

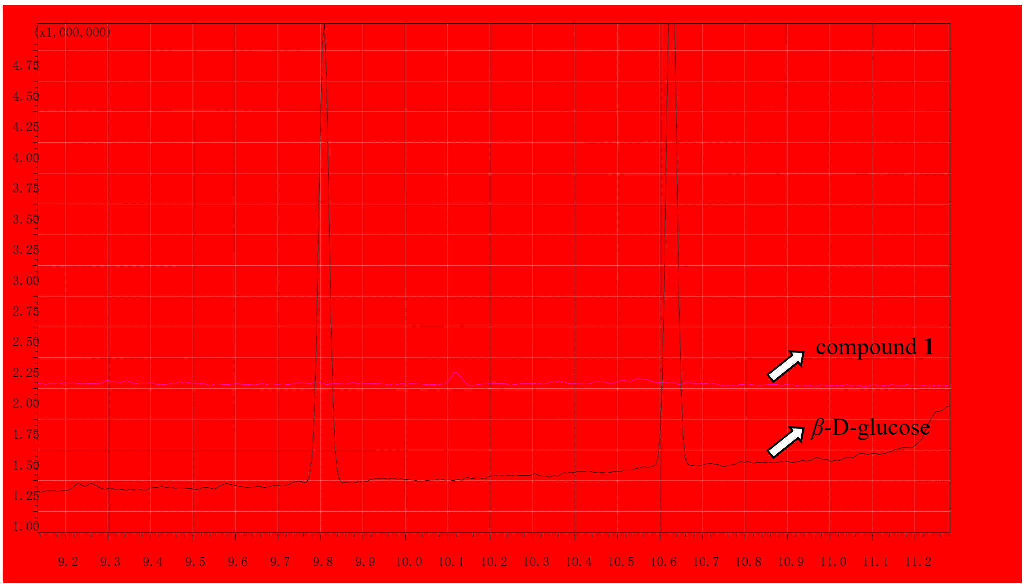

Figure S10. GC-MS spectrum of compound **1**

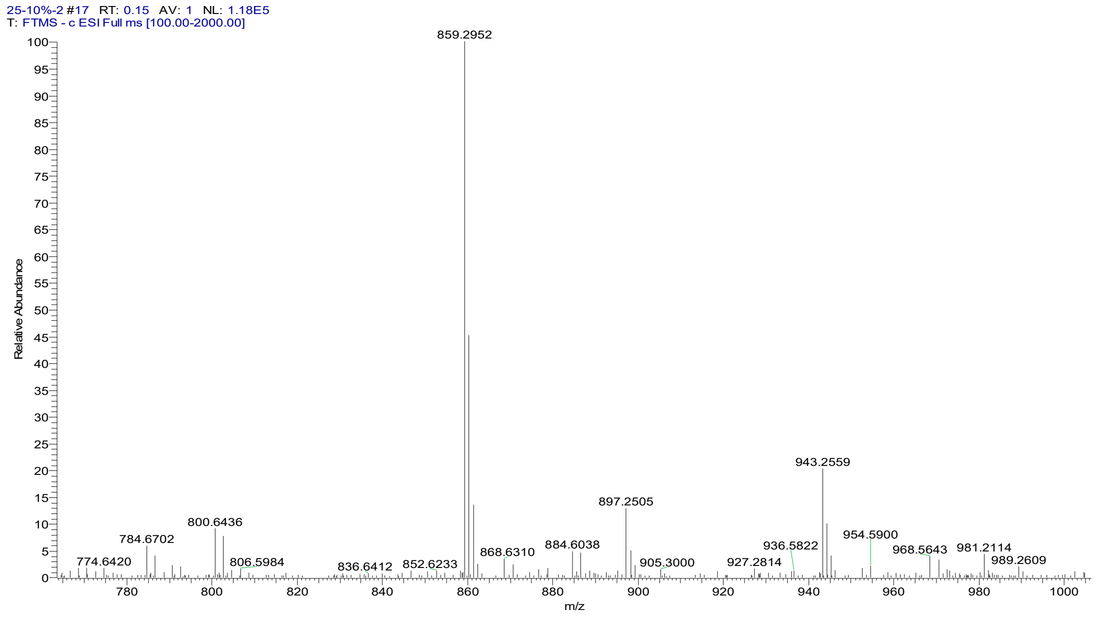

Figure S11. HRESIMS spectrum of compound **2**

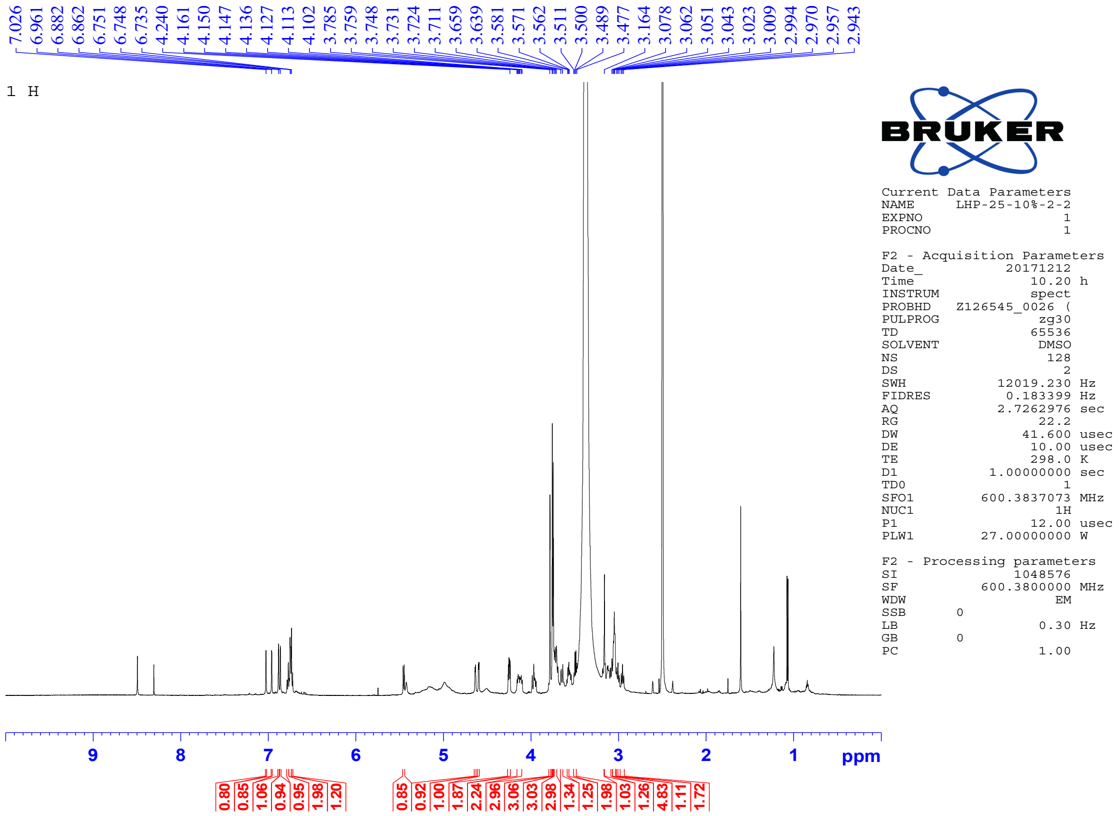

Figure S12. ^{1}H NMR spectrum of compound **2** (DMSO-d_6, 500MHz)

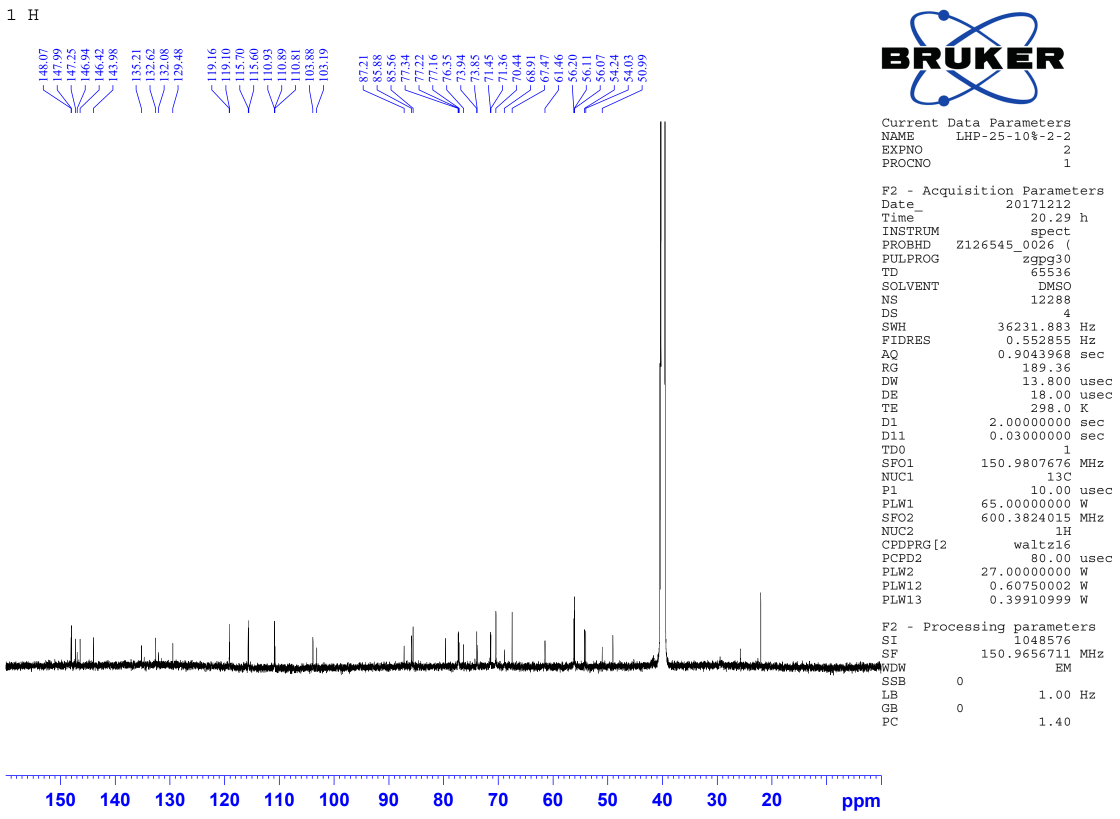

Figure S13. ^{13}C NMR spectrum of compound **2** (DMSO-d_6, 125MHz)

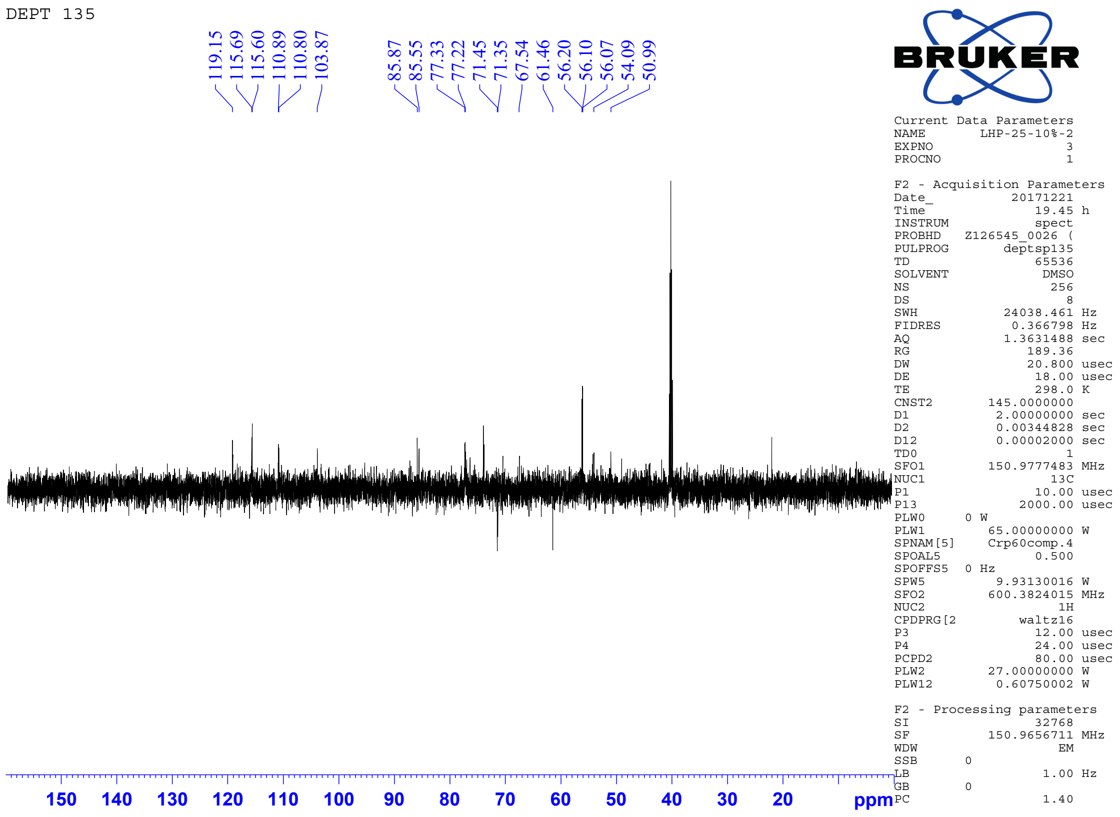

Figure S14. DEPT135 spectrum of compound **2** (DMSO-d_6, 125MHz)

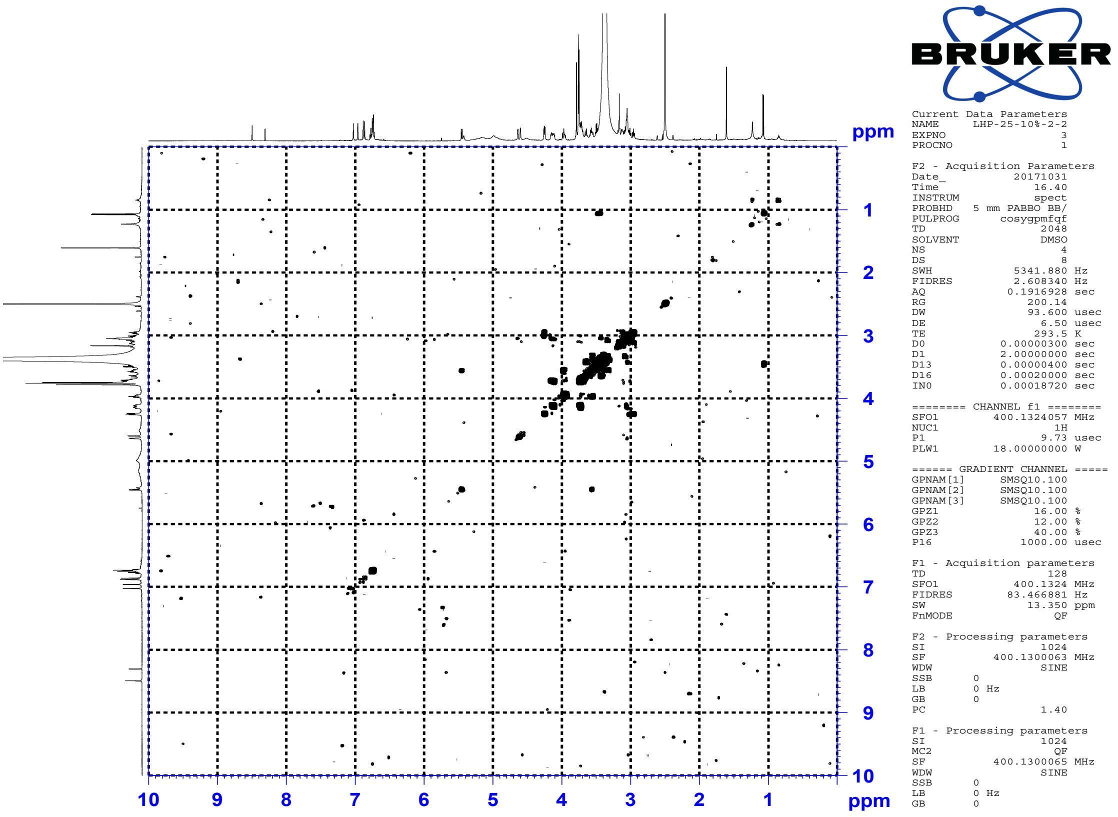

Figure S15. ^{1}H-^{1}H COSY spectrum of compound **2** (DMSO-d_6, 500MHz)

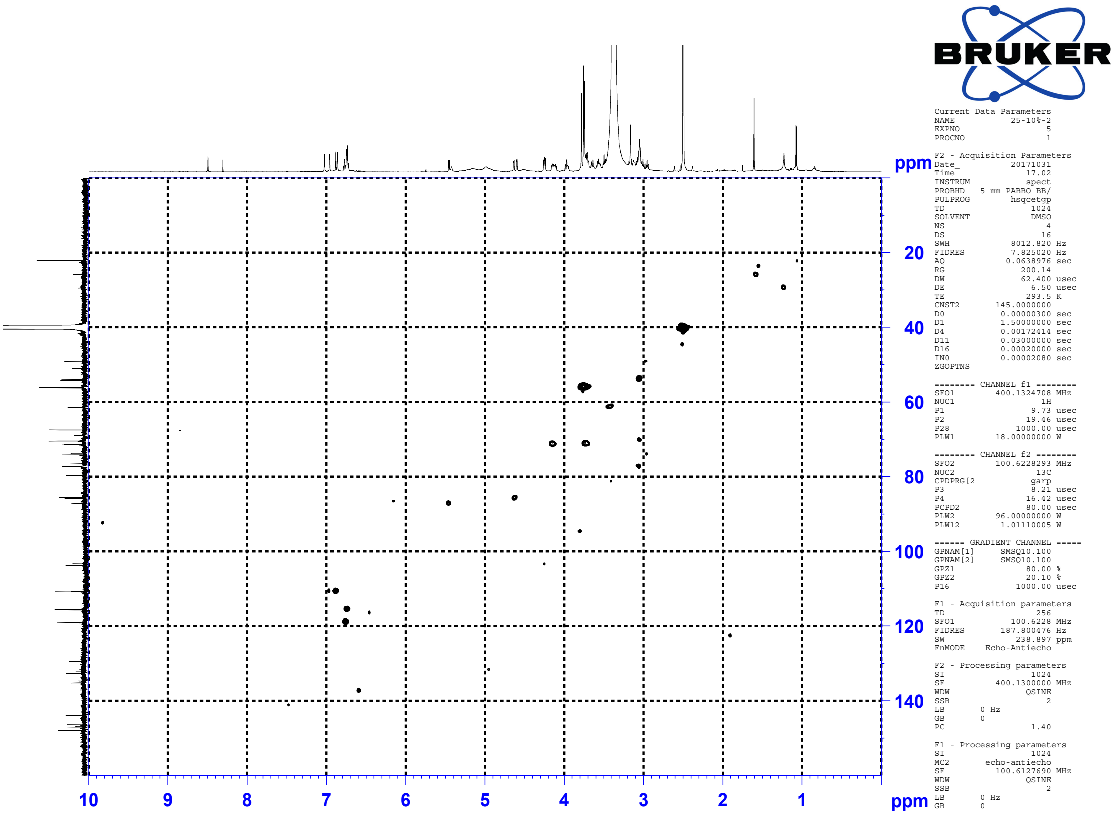

Figure S16. HSQC spectrum of compound **2** (DMSO-d_6, 500MHz)

F

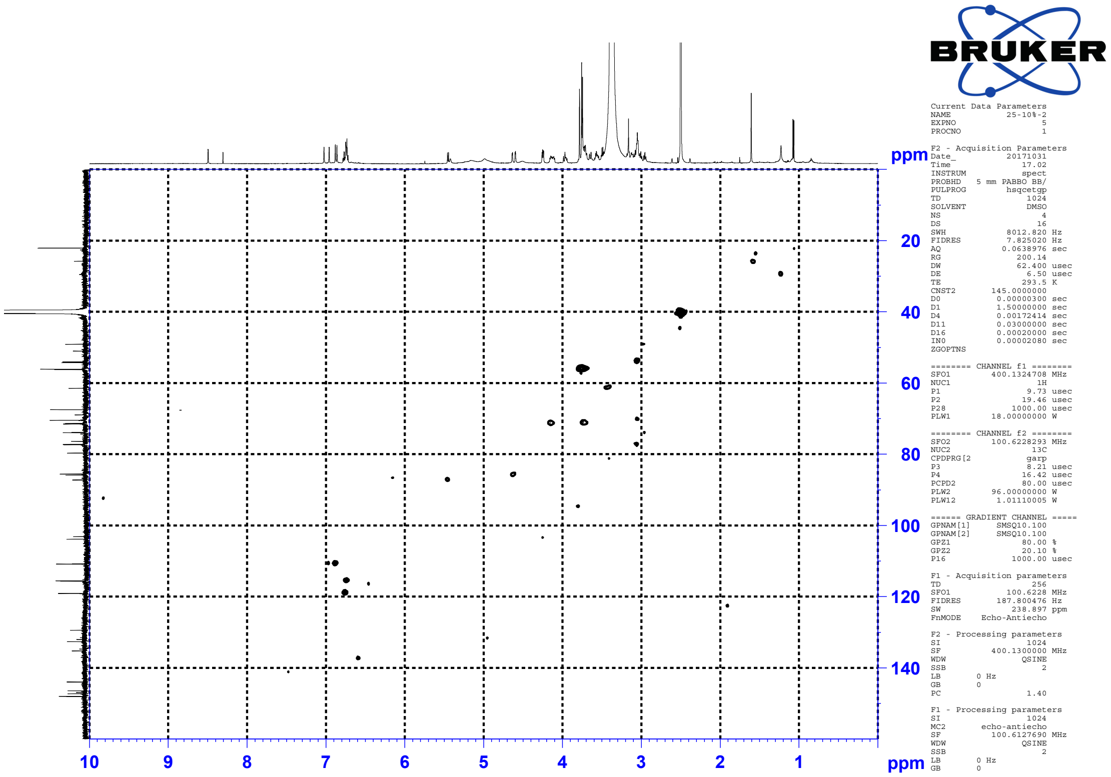

Figure S17. HMBC spectrum of compound **2** (DMSO-d_6, 500MHz)

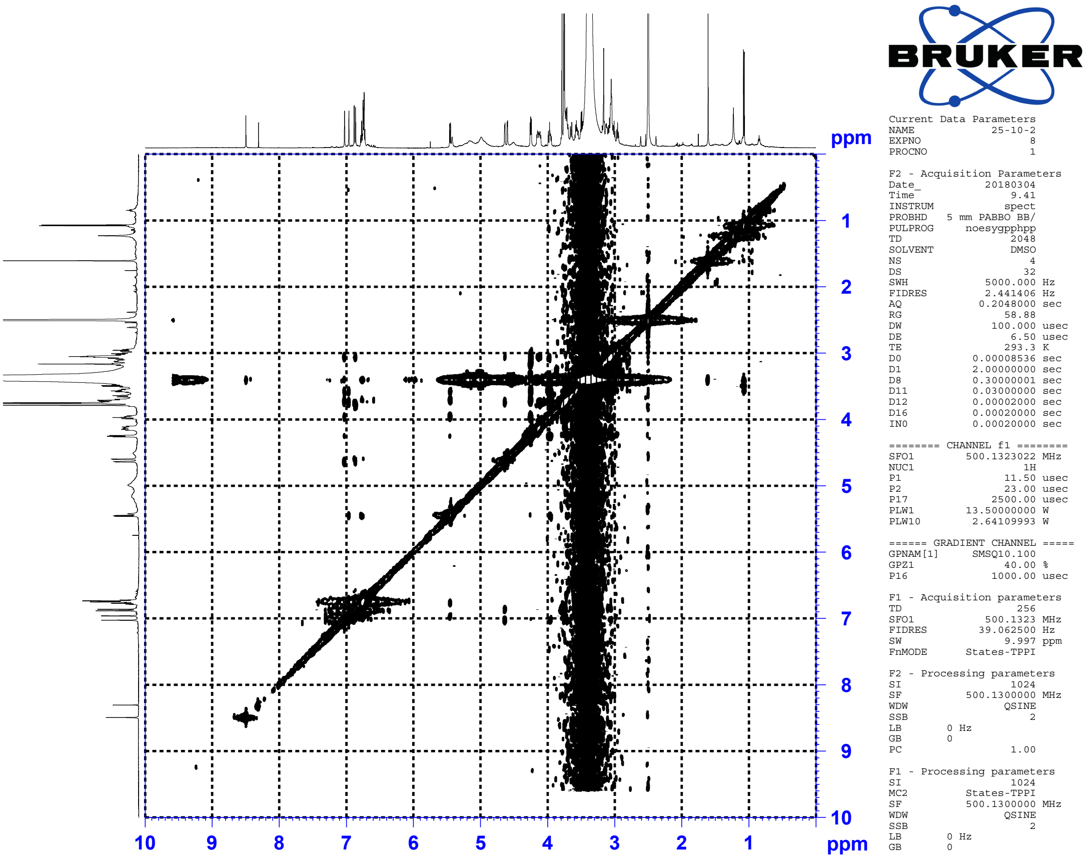

Figure S18. NOESY spectrum of compound **2** (DMSO-d_6, 500MHz)

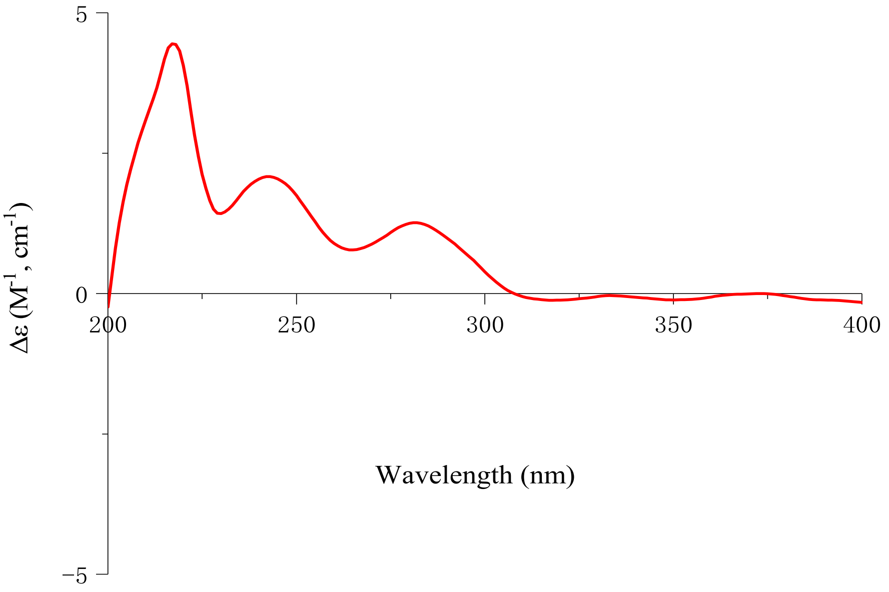

Figure S19. CD spectrum of compound **2** (in MeOH)

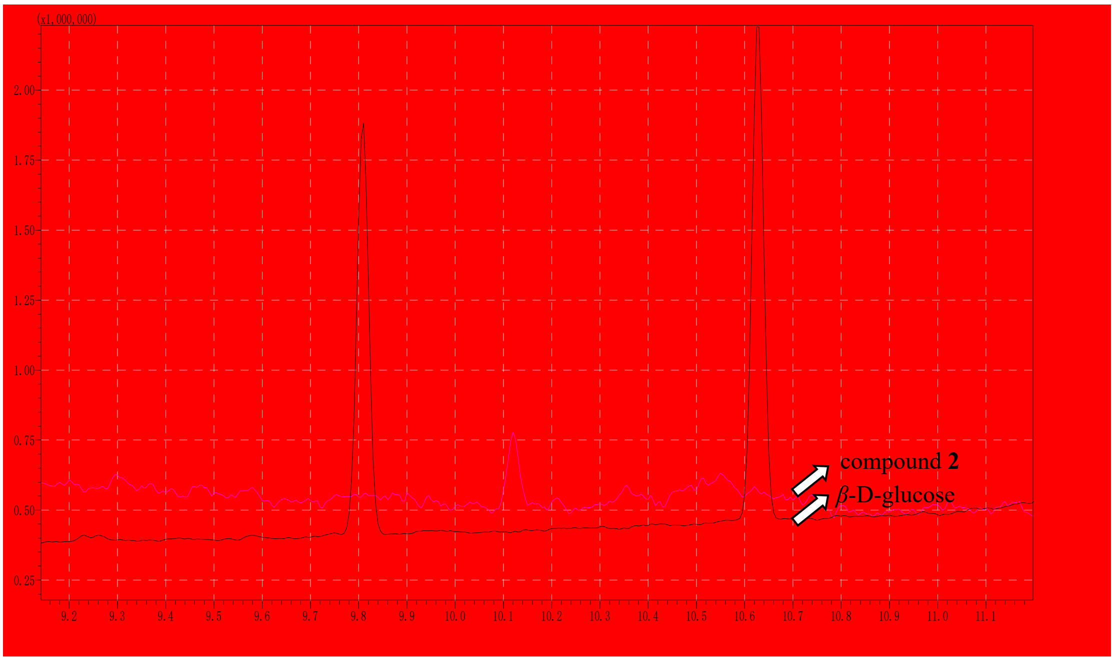

Figure S20. GC-MS spectrum of compound **2**

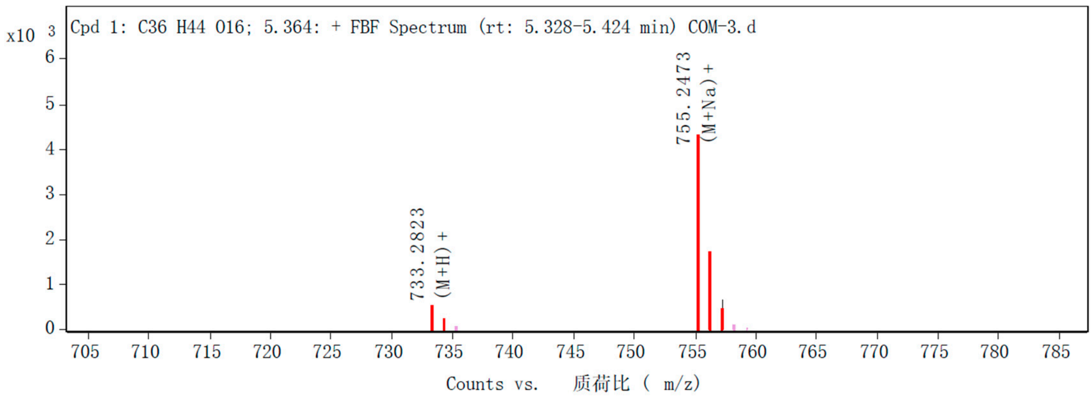

Figure S21. HRESIMS spectrum of compound **3**

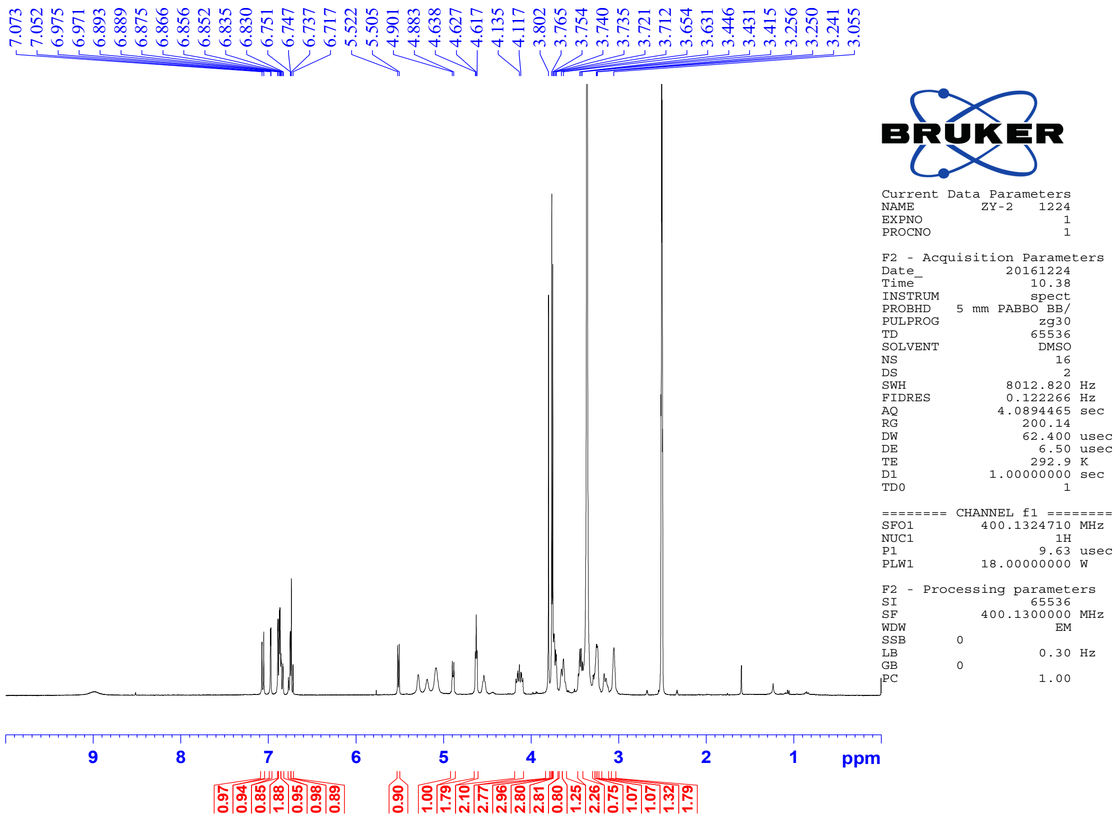

Figure S22. ^{1}H NMR spectrum of compound **3** (DMSO-d_6, 500MHz)

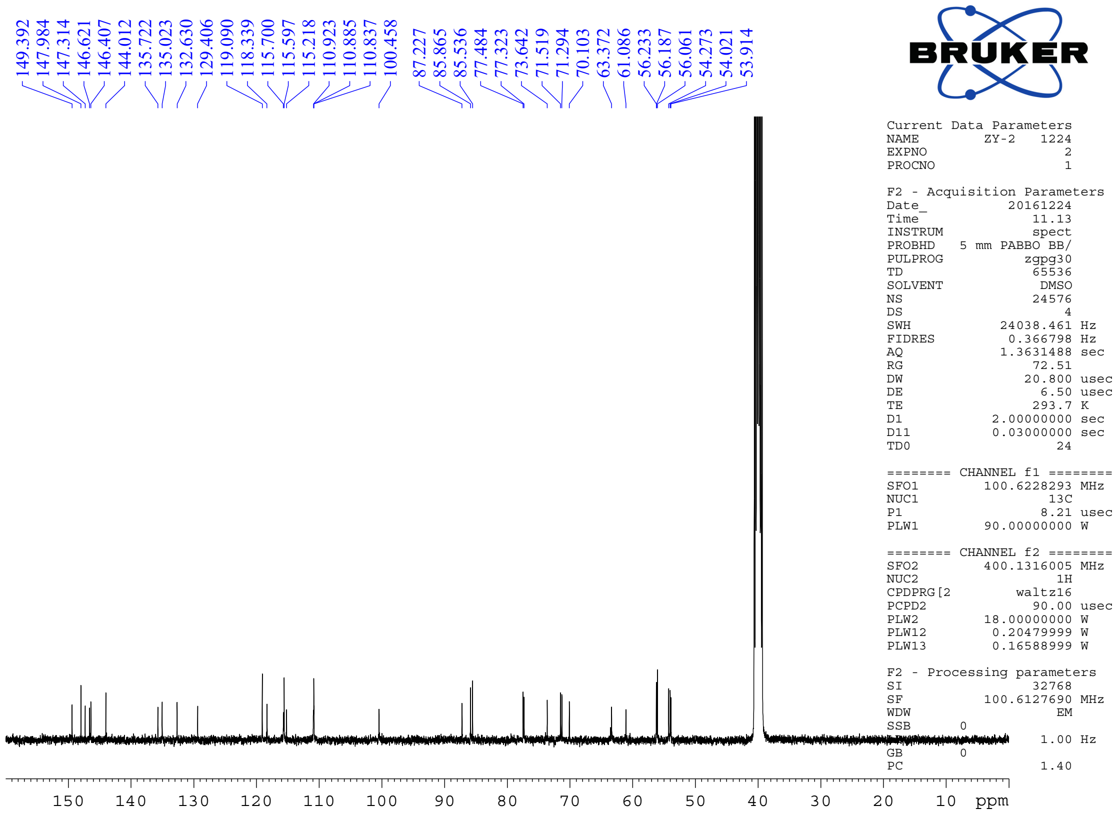

Figure S23. ^{13}C NMR spectrum of compound **3** (DMSO-d_6, 125MHz)

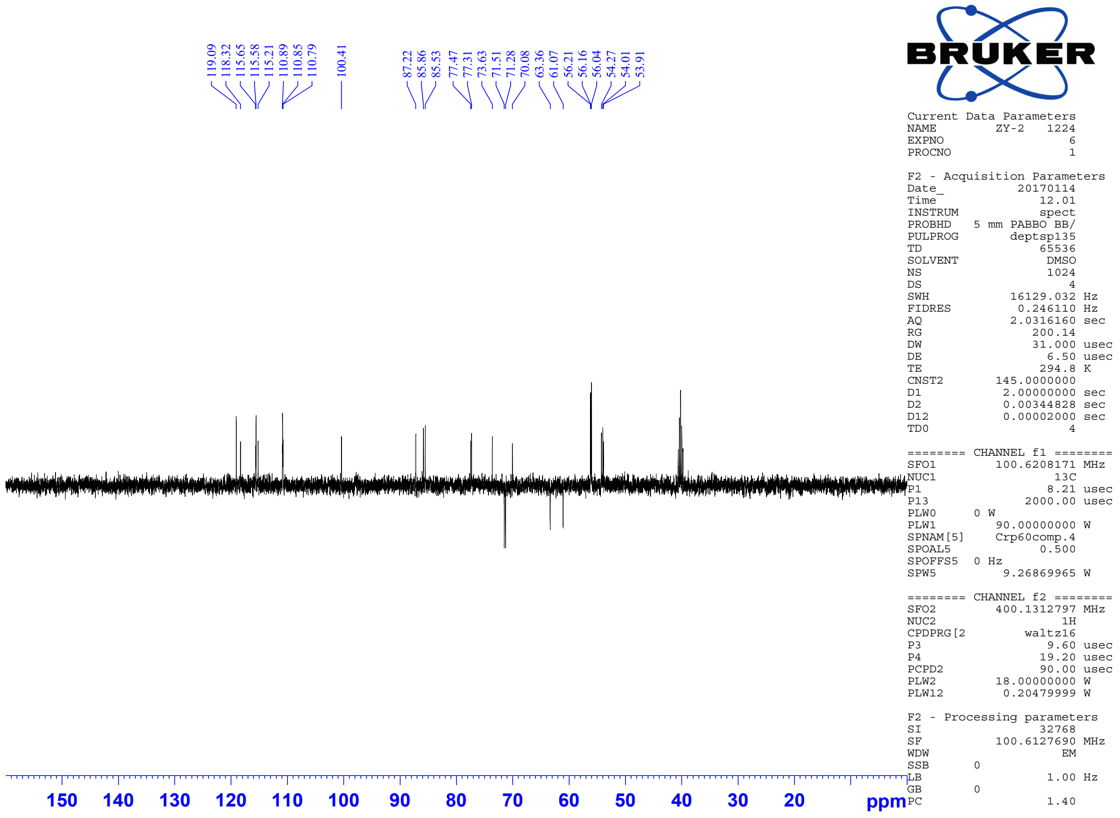

Figure S24. DEPT135 spectrum of compound **3** (DMSO-d_6, 125MHz)

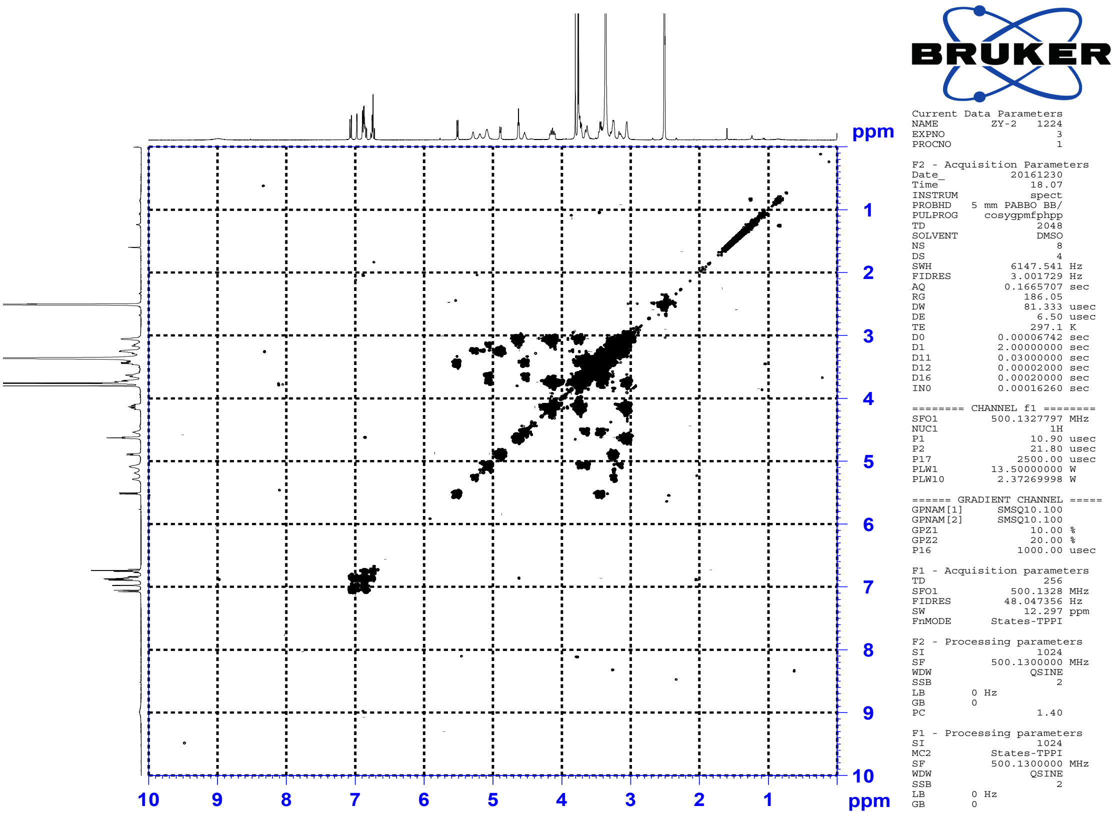

Figure S25. ^{1}H-^{1}H COSY spectrum of compound **3** (DMSO-d_6, 500MHz)

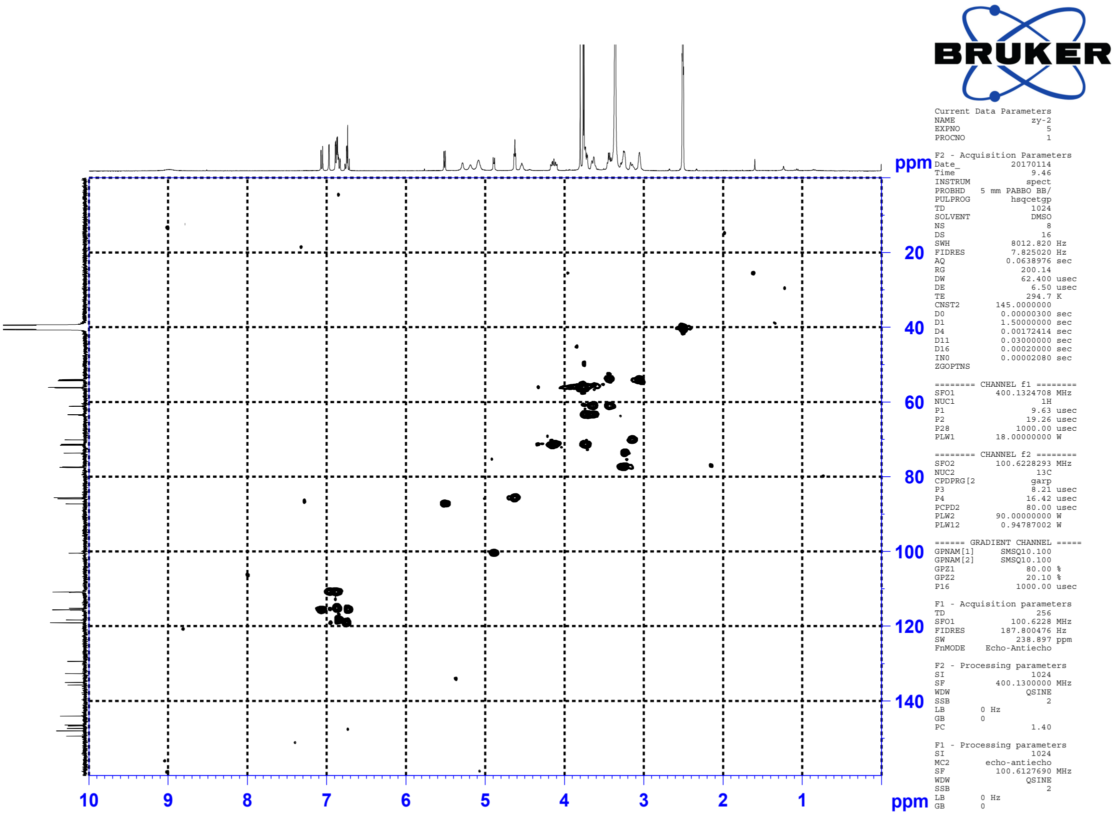

Figure S26. HSQC spectrum of compound **3** (DMSO-d_6, 500MHz)

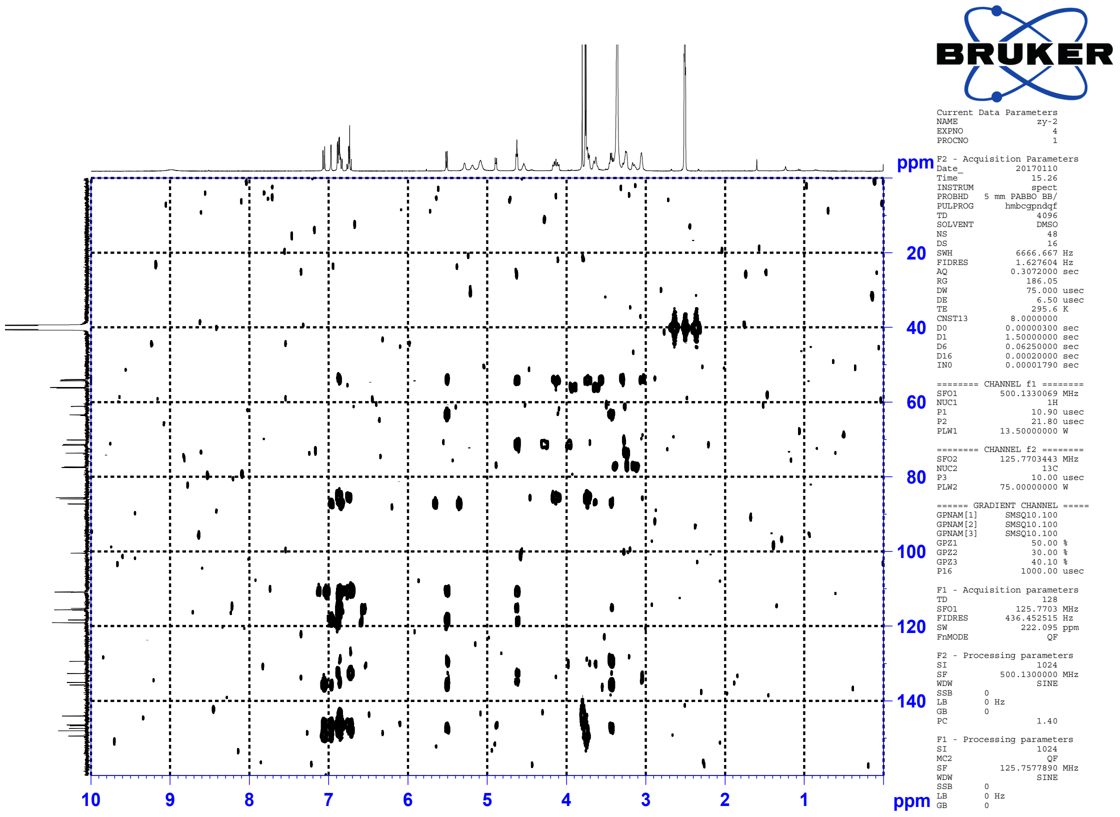

Figure S27. HMBC spectrum of compound **3** (DMSO-*d*$_6$, 500MHz)

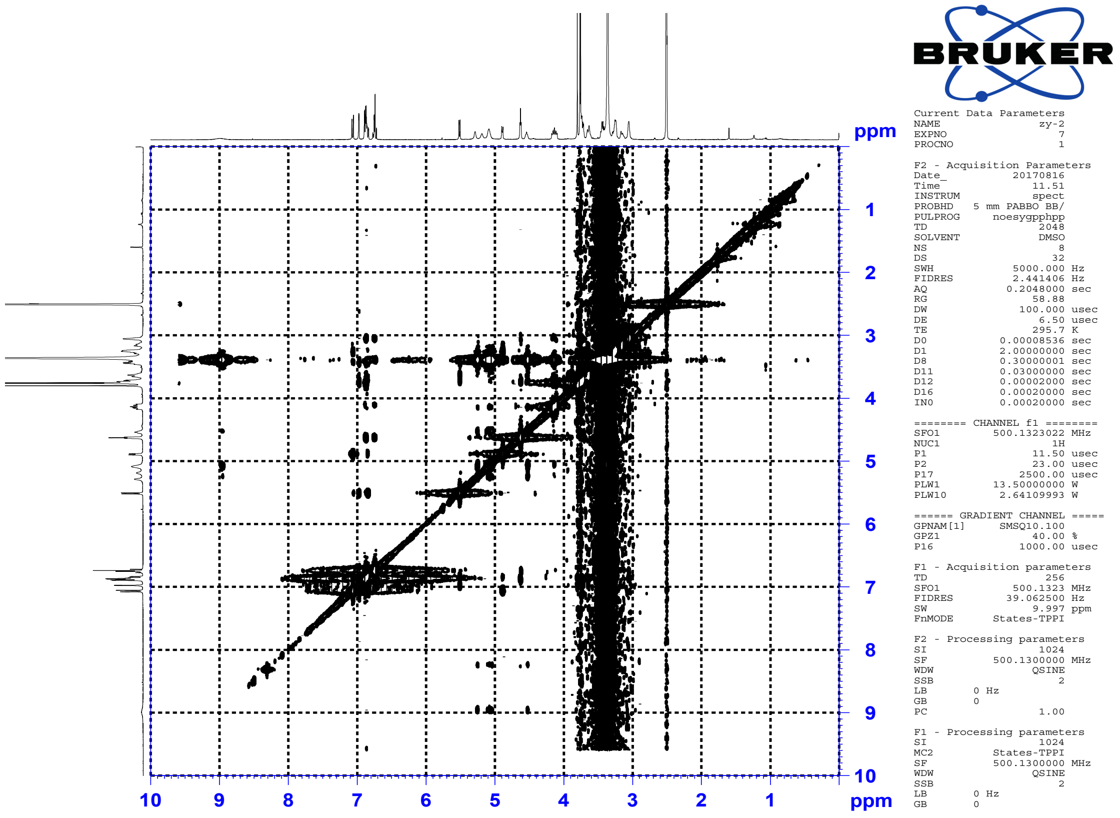

Figure S28. NOESY spectrum of compound **3** (DMSO-d6, 500MHz)

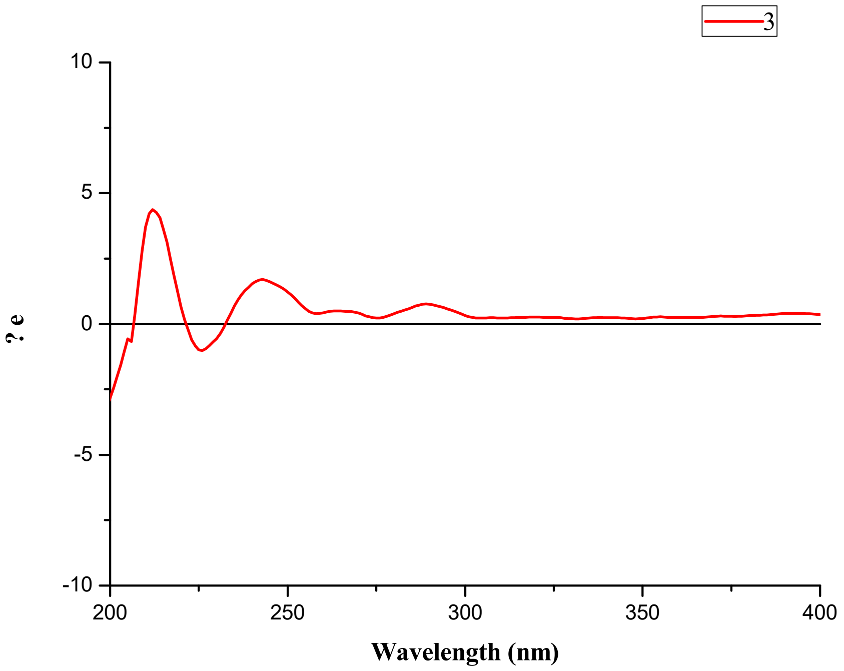

Figure S29. CD spectrum of compound **3** (in MeOH)

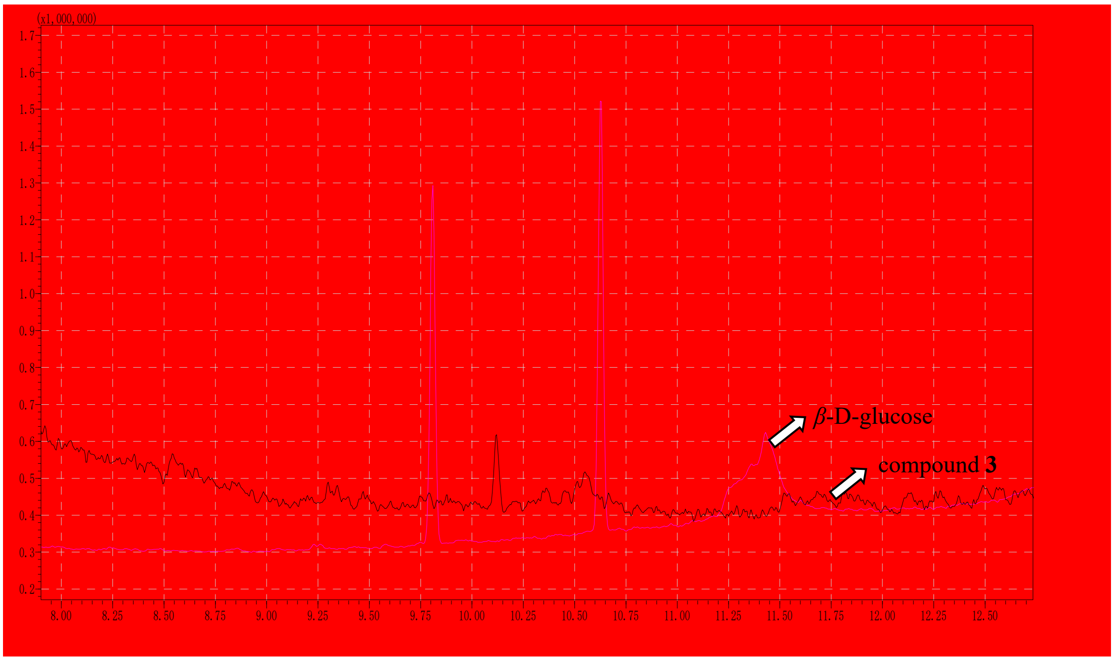

Figure S30. GC-MS spectrum of compound **3**

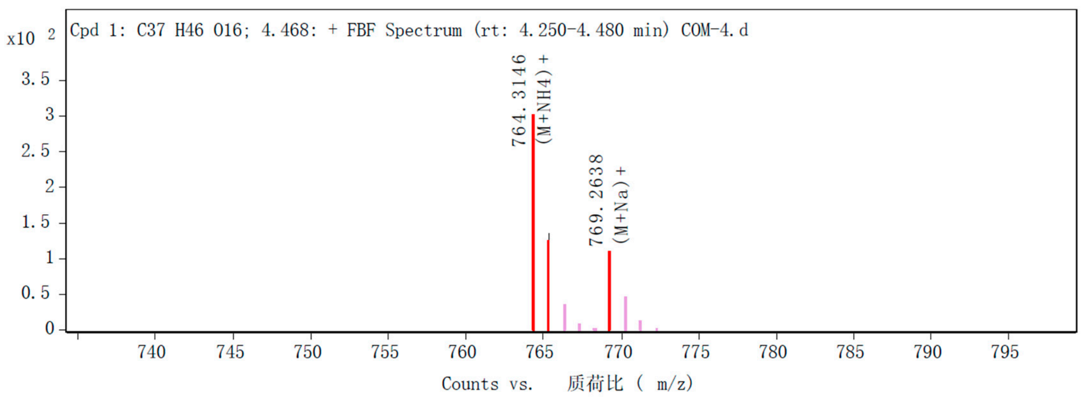

Figure S31. HRESIMS spectrum of compound **4**

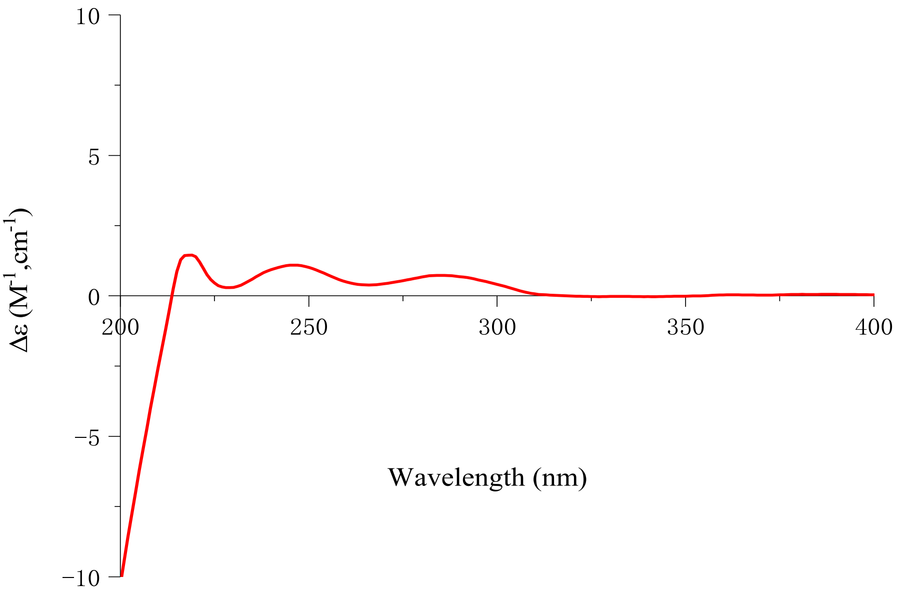

Figure S32. CD spectrum of compound **4** (in MeOH)

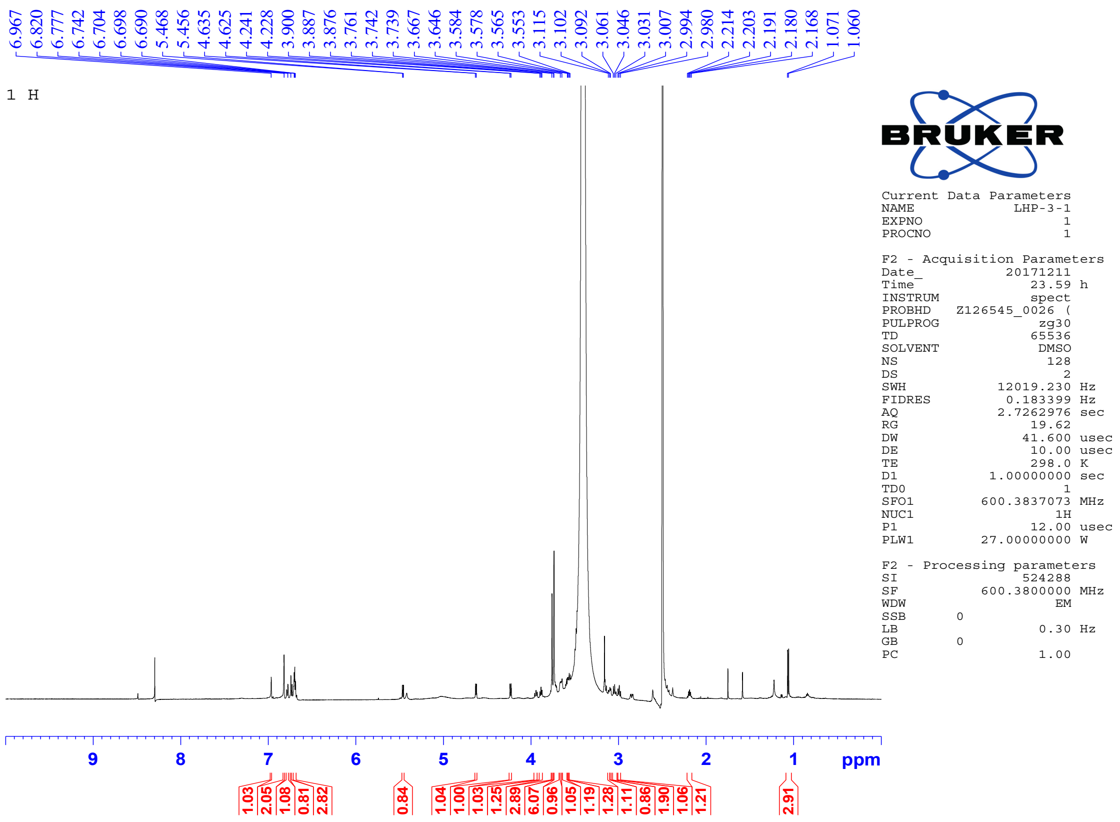

Figure S33. ^{1}H NMR spectrum of compound **4** (DMSO-d_6, 500MHz)

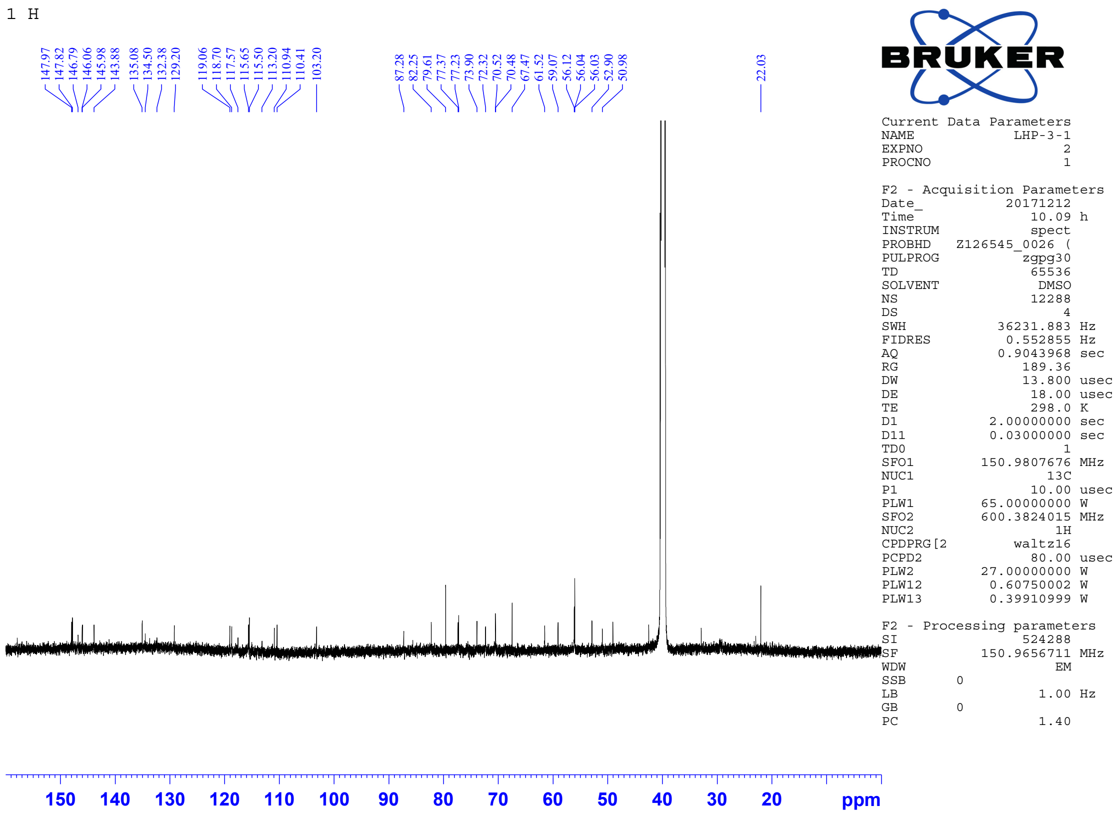

Figure S34. ^{13}C NMR spectrum of compound **4** (DMSO-d_6, 125MHz)

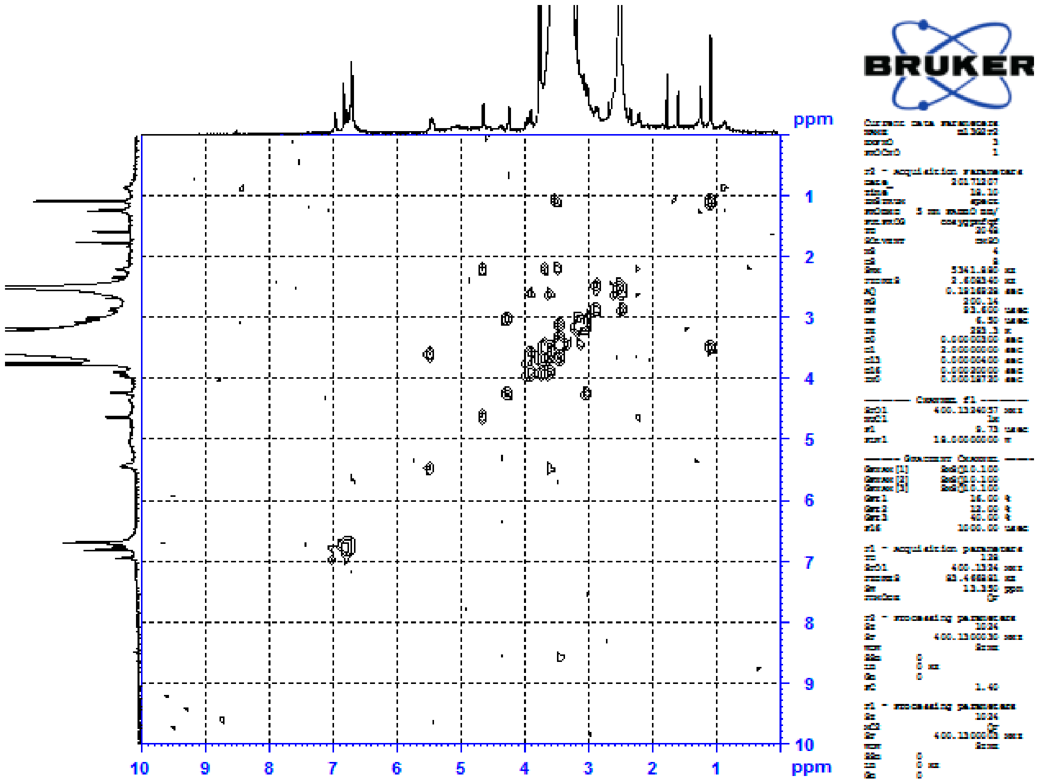

Figure S35. ^{1}H-^{1}H COSY spectrum of compound **4** (DMSO-d_6, 500MHz)

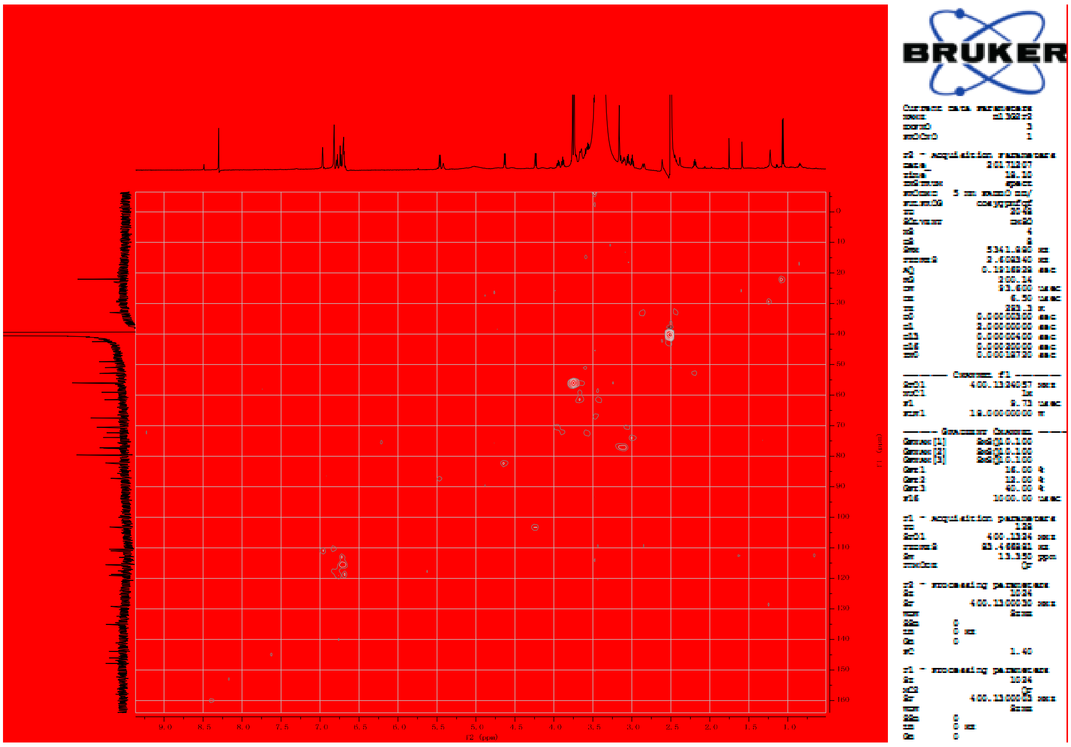

Figure S36. HSQC spectrum of compound **4** (DMSO-d_6, 500MHz)

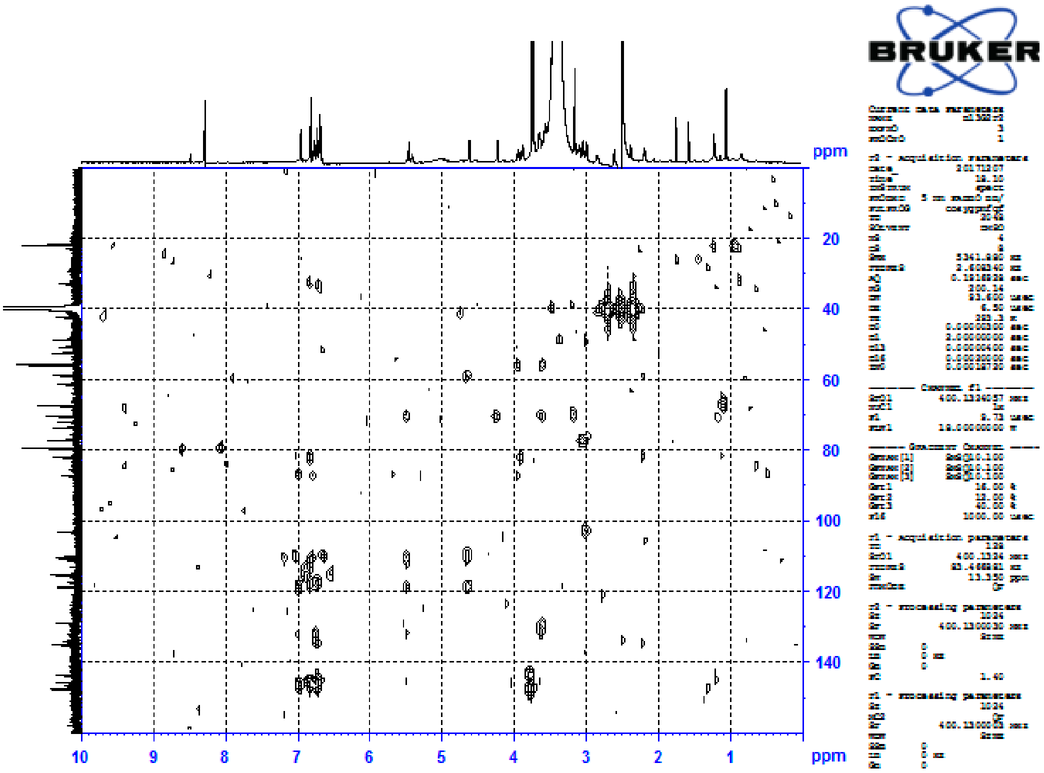

Figure S37. HMBC spectrum of compound **4** (DMSO-d_6, 500MHz)

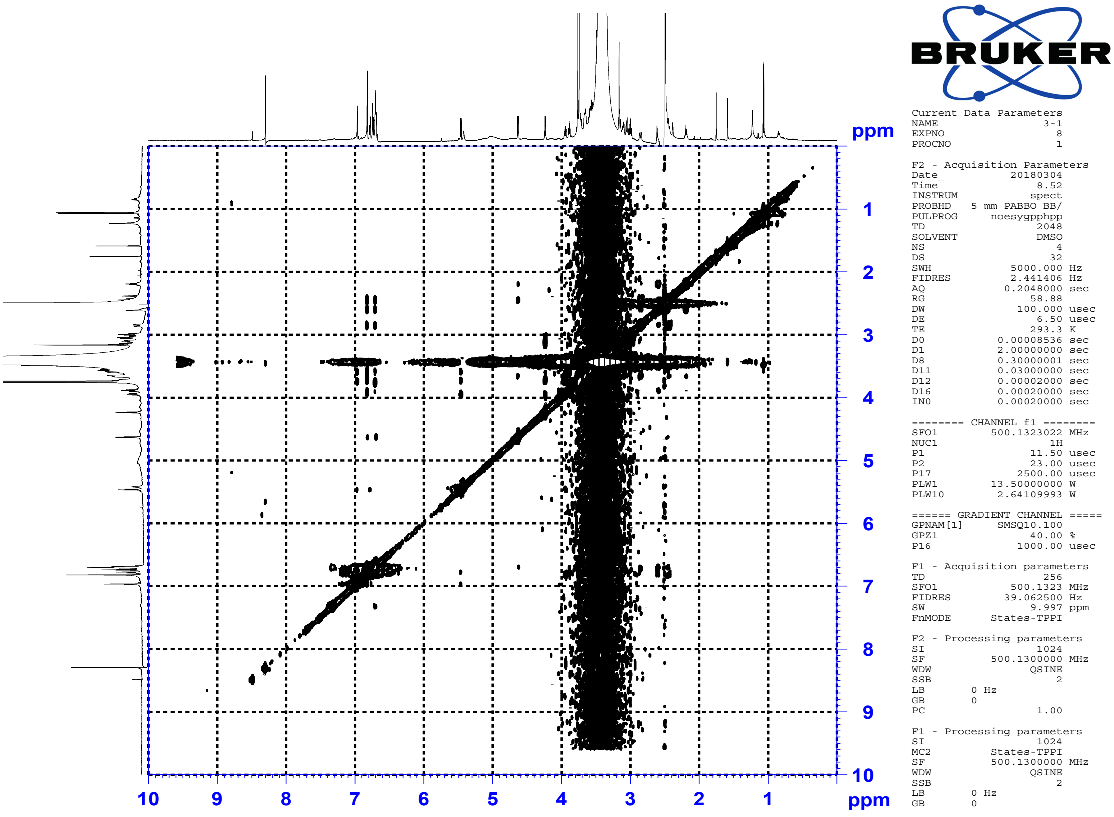

Figure S38. NOESY spectrum of compound **4** (DMSO-d_6, 500MHz)

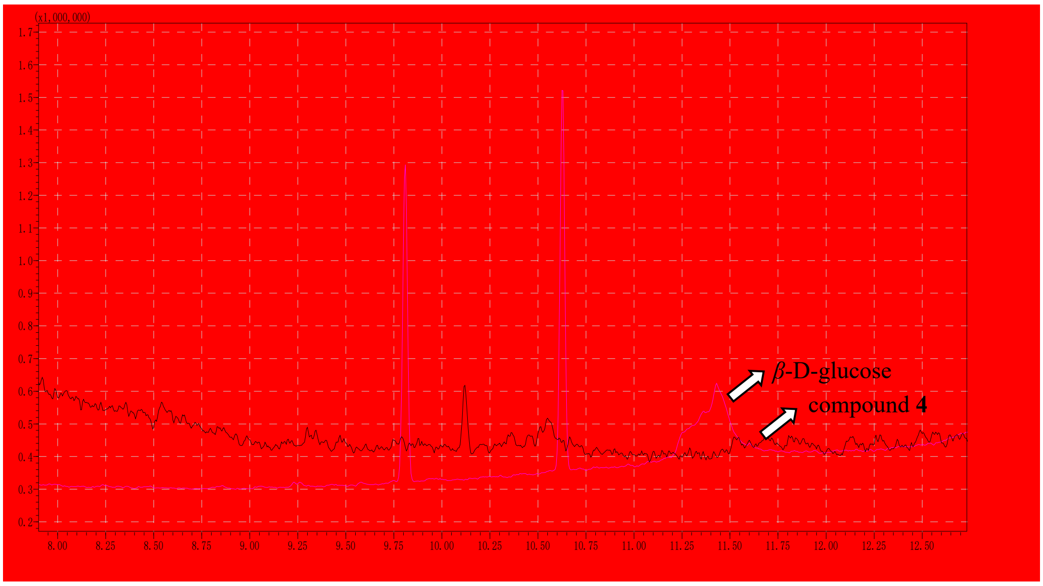

Figure S39. GC-MS spectrum of compound **4**

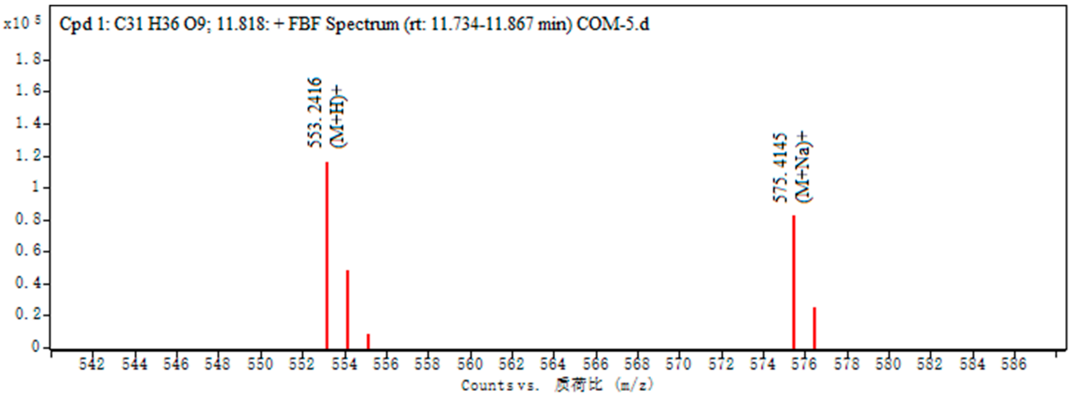

Figure S40. HRESIMS spectrum of compound **5**

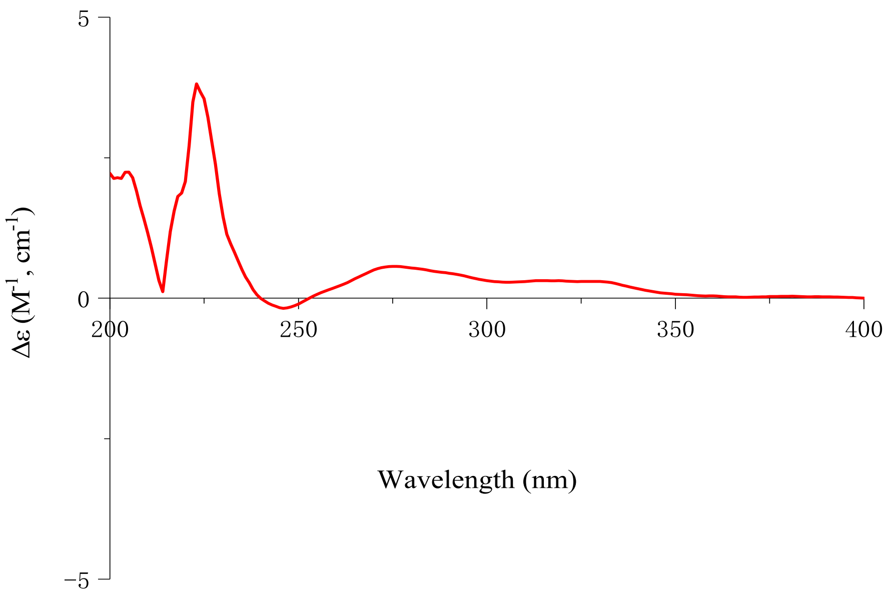

Figure S41. CD spectrum of compound **5** (in MeOH)

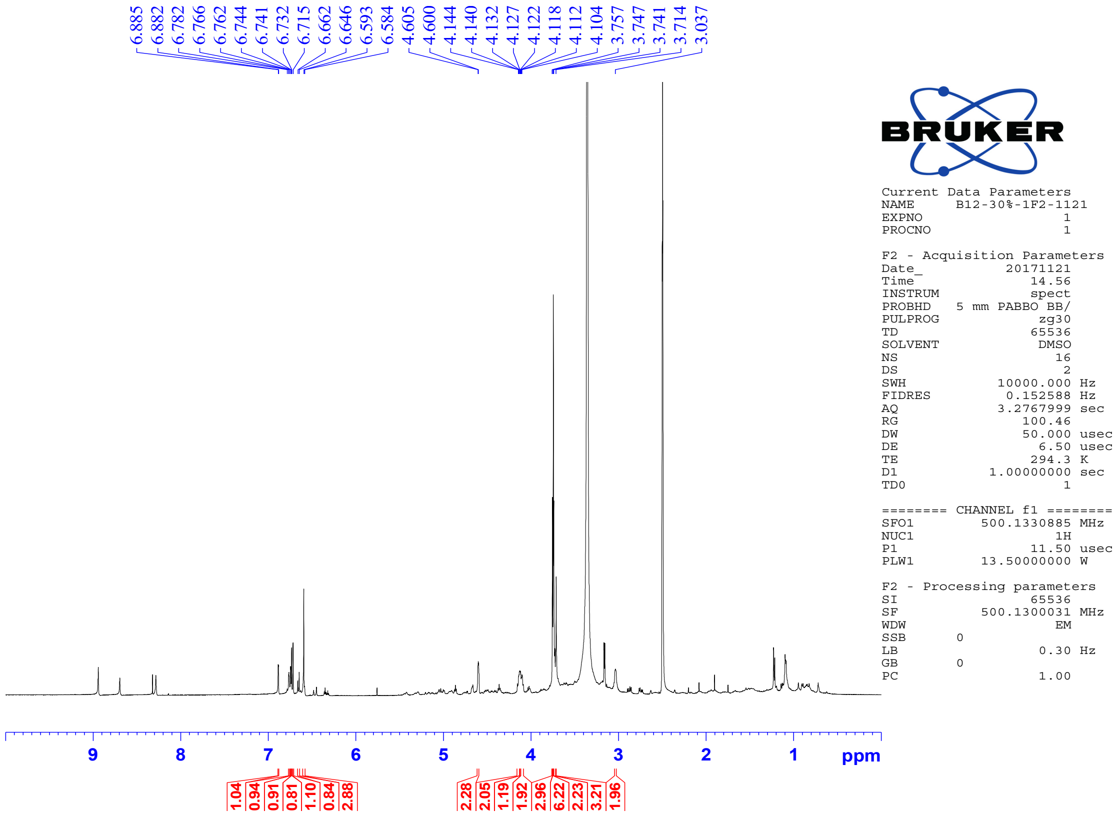

Figure S42. ^{1}H NMR spectrum of compound **5** (DMSO-d_6, 500MHz)

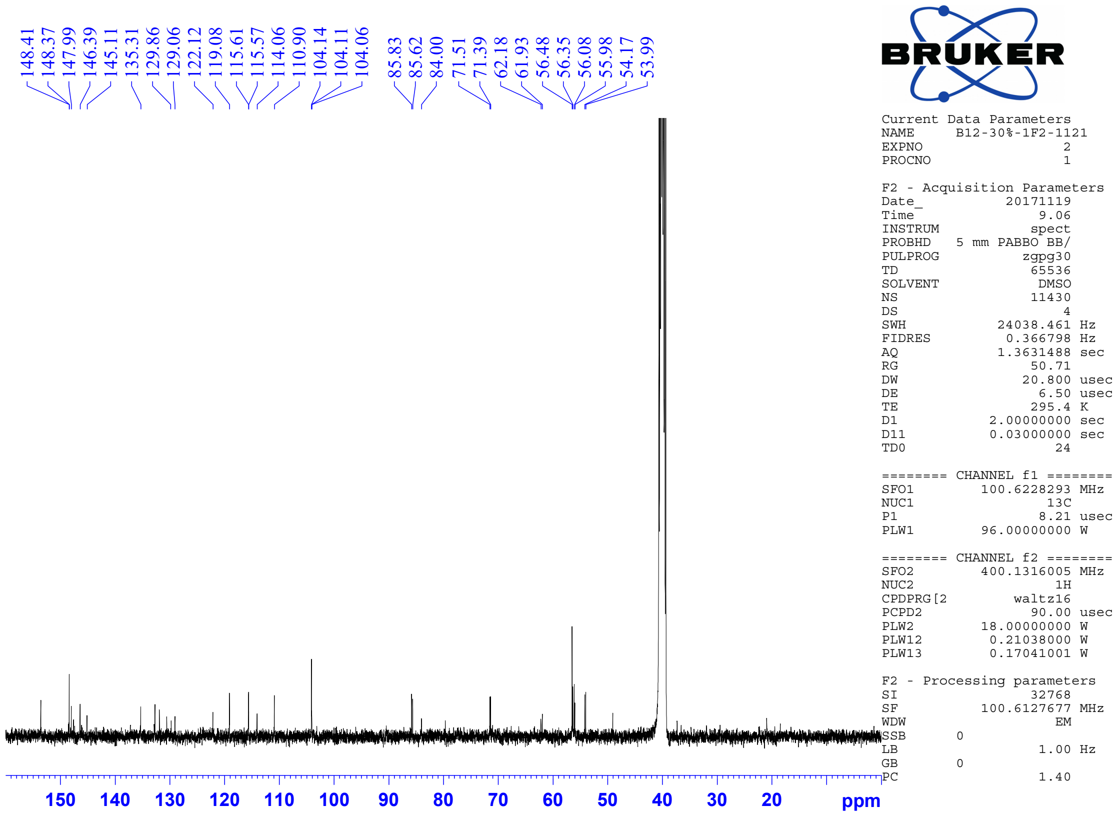

Figure S43. ^{13}C NMR spectrum of compound **5** (DMSO-d_6, 125MHz)

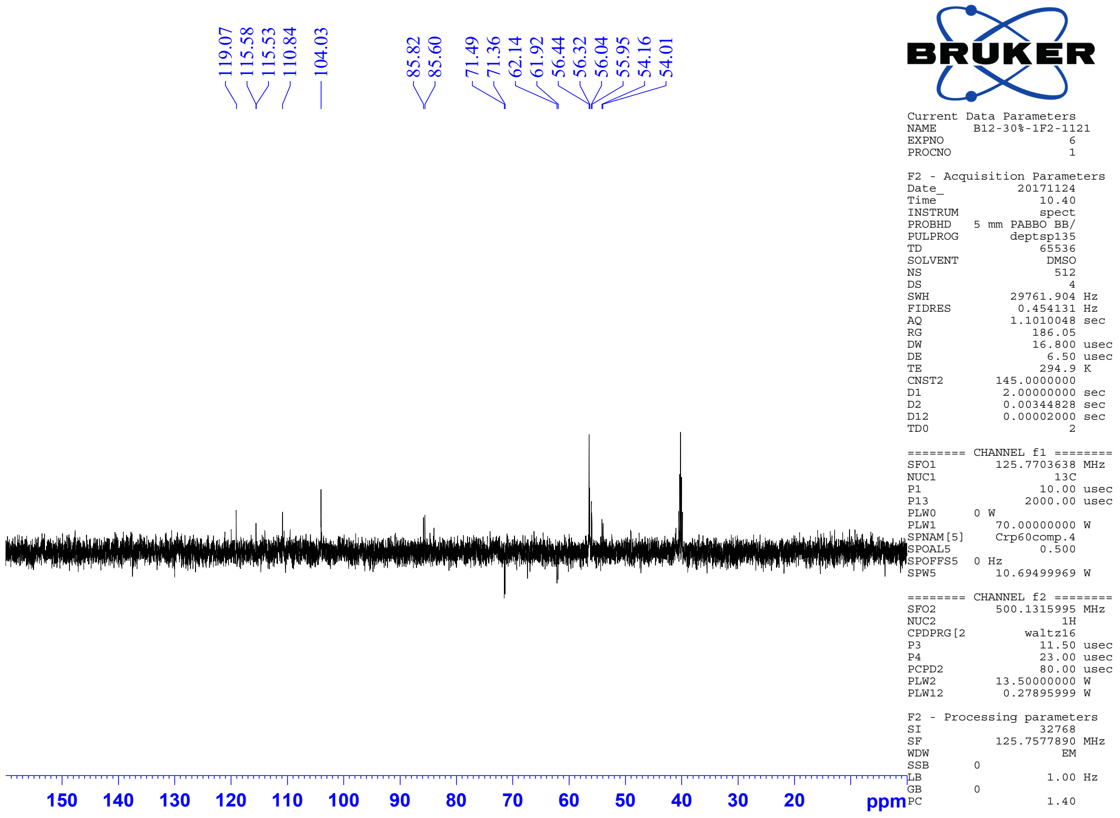

Figure S44. DEPT135 spectrum of compound **5** (DMSO-d_6, 125MHz)

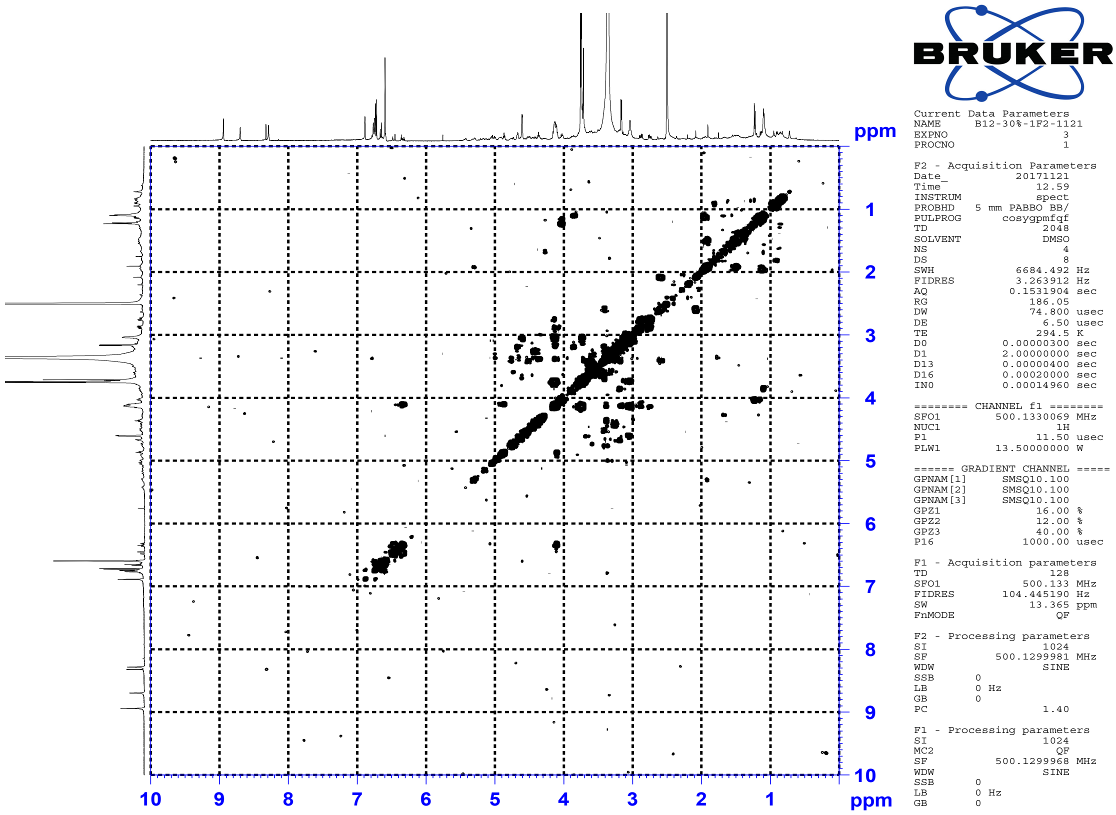

Figure S45. ^{1}H-^{1}H COSY spectrum of compound **5** (DMSO-d_6, 500MHz)

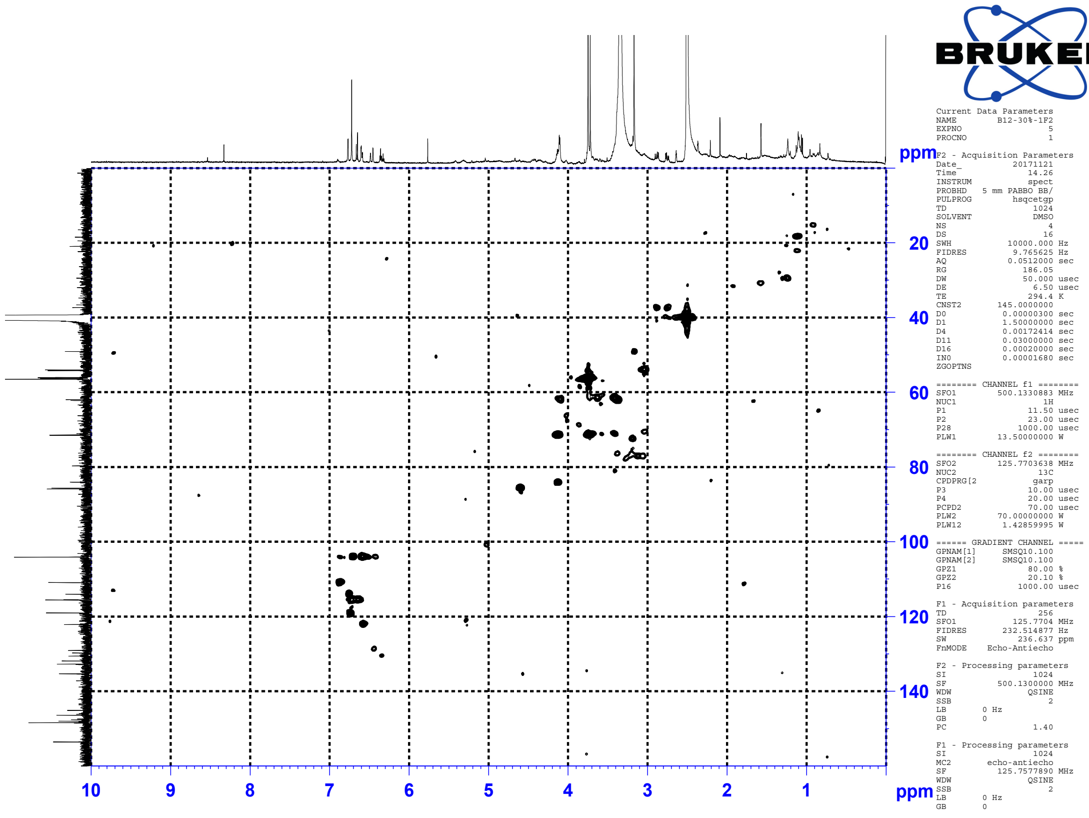

Figure S46. HSQC spectrum of compound **5** (DMSO-d_6, 500MHz)

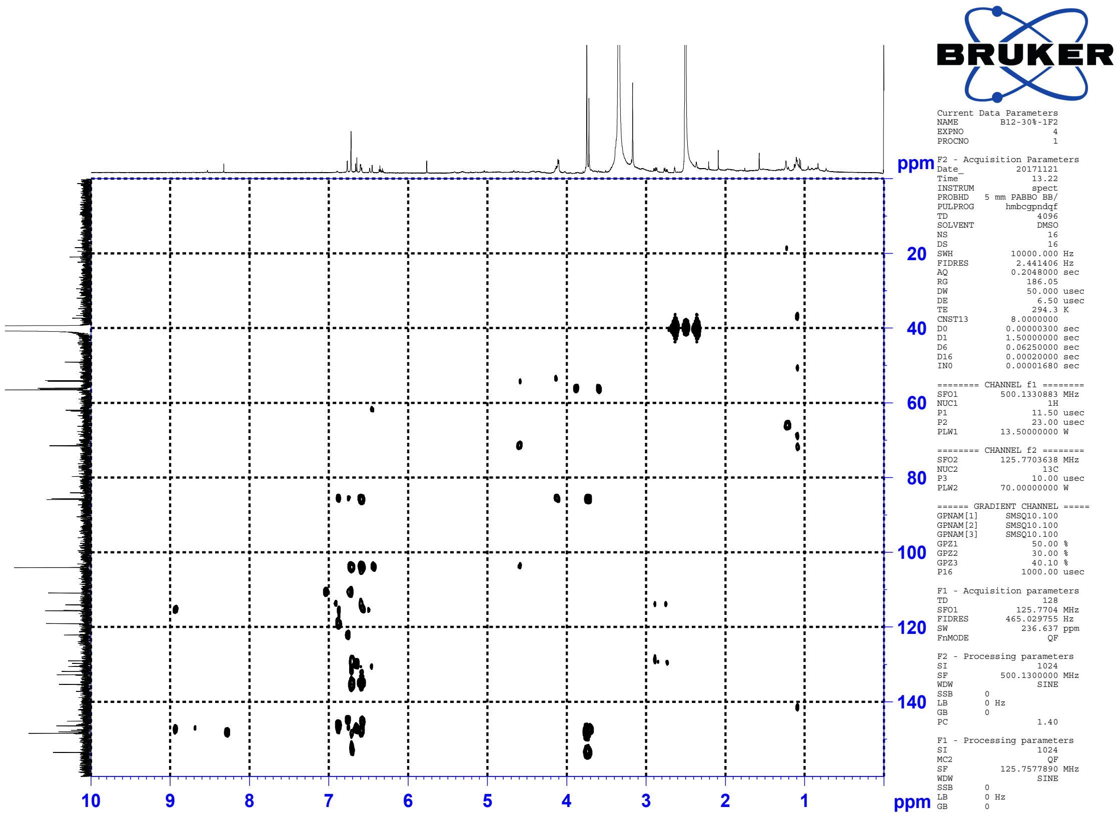

Figure S47. HMBC spectrum of compound **5** (DMSO-d_6, 500MHz)

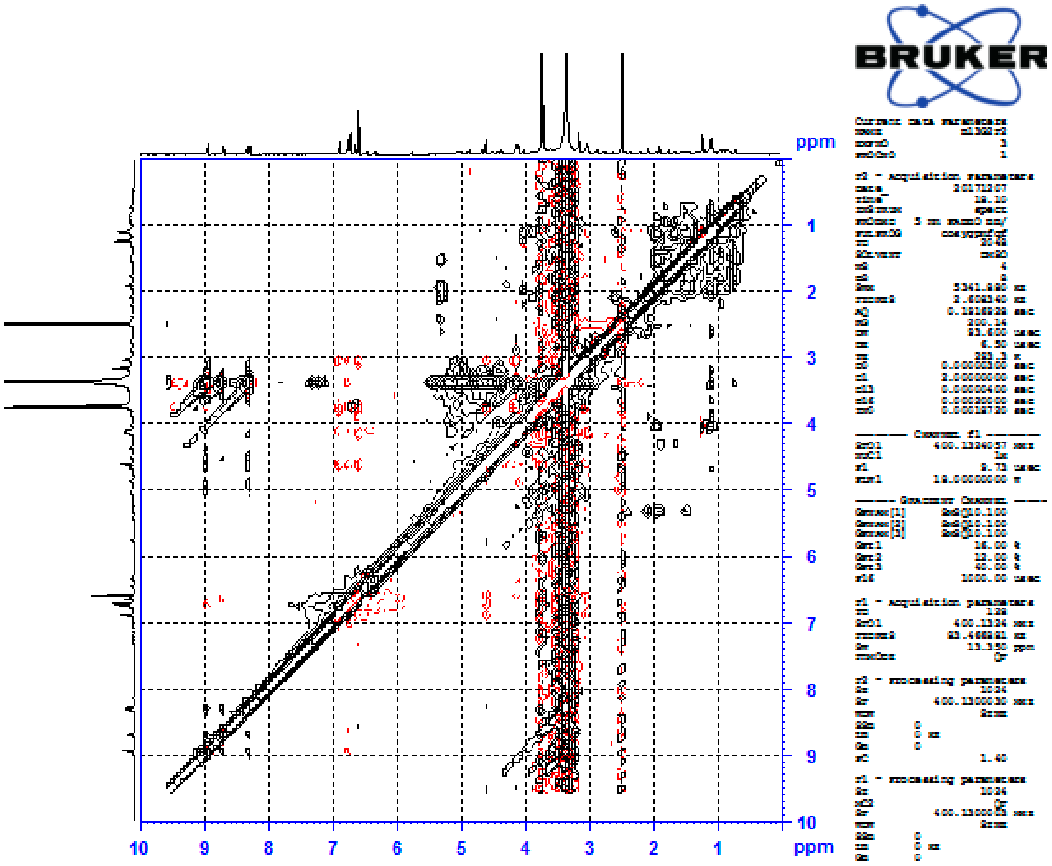

Figure S48. NOESY spectrum of compound **5** (DMSO-d_6, 500MHz)
